# Supplementary material for: Fractionating Blunted Reward Processing Characteristic of Anhedonia by Over-Activating Primate Subgenual Anterior Cingulate Cortex
Source: Neuron. 2019 Jan 16;101(2):307–320.e6. doi: 10.1016/j.neuron.2018.11.021 (PMC6344231; doi:10.1016/j.neuron.2018.11.021)
Supplement: Document S1. Figures S1–S8 and Tables S1–S8 [file mmc1.pdf]

Neuron, Volume *101*

Supplemental Information

Fractionating Blunted Reward Processing Characteristic of Anhedonia by Over-Activating Primate Subgenual Anterior Cingulate Cortex

Laith Alexander, Philip L.R. Gaskin, Stephen J. Sawiak, Tim D. Fryer, Young T. Hong, Gemma J. Cockcroft, Hannah F. Clarke, and Angela C. Roberts

Supplemental

Figures

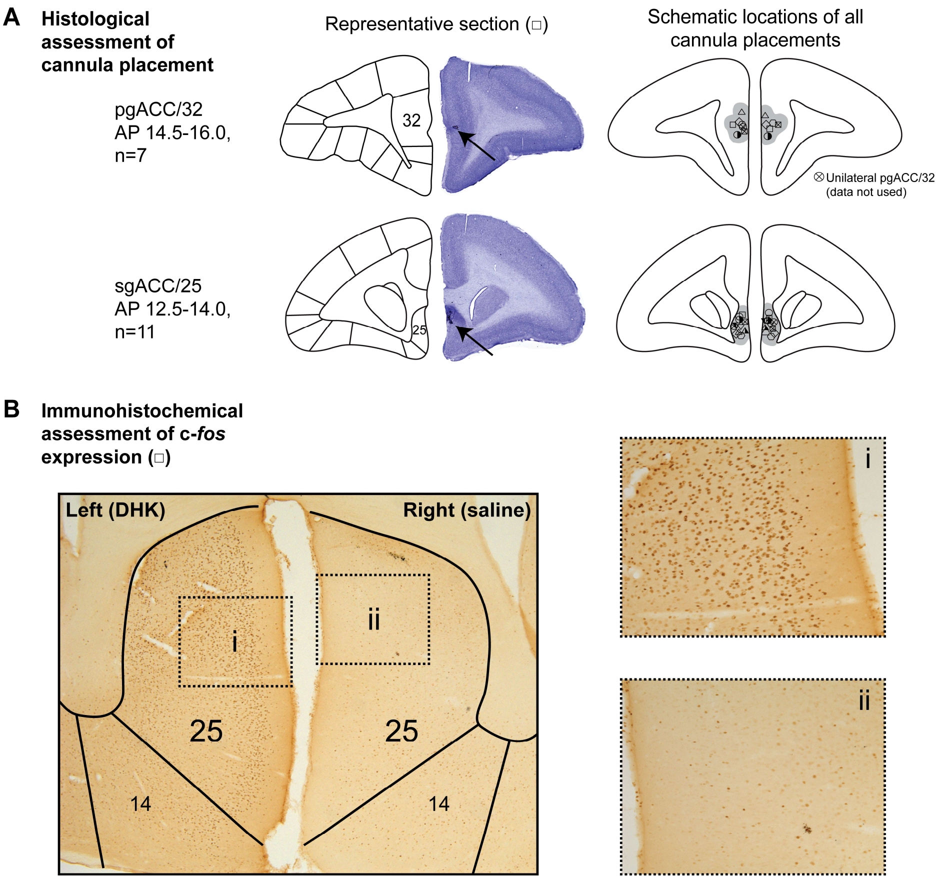

Figure S1. Verification of cannula placements and immunohistochemical assessment of c-*fos* expression. Related to Table 1 and all figures. A Histological assessment of cannula placement using cresyl violet staining. Representative sections are shown with pgACC/32 and sgACC/25 cannulation sites indicated by an arrow. No damage was noted in any animals apart from the small area of gliosis used to pinpoint cannula placement. A schematic diagram shows the cannula placements for all monkeys reported in the manuscript together with estimated infusion spread in grey (approximately 0.5-1.0mm). **B** c-*fos* expression was assessed at the end of the study in one marmoset (□) following DHK infusion in left sgACC/25 and saline infusion in right sgACC/25. DHK infusions (inset i) – but not saline infusions (inset ii) – caused robust c-*fos* expression in sgACC/25. This subject had the highest number of infusions out of all subjects reported in this manuscript, showing that the cortex remains sensitive to drug manipulations.

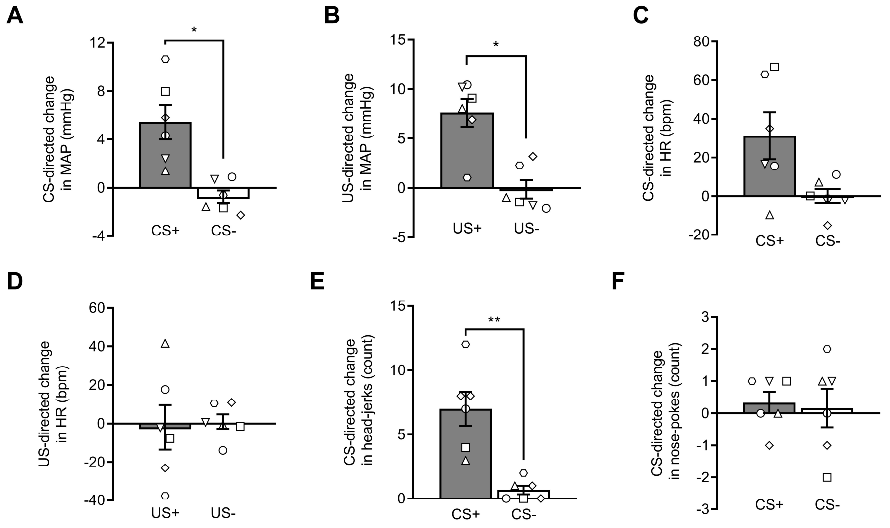

Figure S2. **Cardiovascular and behavioral measures associated with appetitive Pavlovian conditioning. Related to Figure 1.** Relevant graphs show mean ± SEM (n=6). **A** Animals showed CS-directed (CS minus baseline) anticipatory MAP responses to the CS+ but not the CS- (two-tailed paired *t*-test, p=0.013). **B** Animals showed US-directed (US minus CS) consummatory MAP responses to the US+ but not the US- (two-tailed paired *t*-test, p=0.017). **C** Heart rate (HR) discrimination was evident in five of six animals, although the response magnitudes were variable resulting in a non-significant discrimination (two-tailed paired *t*-test, p=0.077). **D** HR responses during the consummatory period were highly variable, with no discrimination evident between US+ and US- (two-tailed paired *t*-test, p=0.848). **E** Behaviorally, animals showed rapid orienting responses (head-jerks) to the CS+ but not the CS- (two tailed paired *t*-test, p=0.003). **F** Nose-pokes were measured as a US-directed conditioned behavior during the CS period. Nose-poking behavior was highly variable and did not discriminate between CS type (two-tailed paired *t*-test, p=0.793).

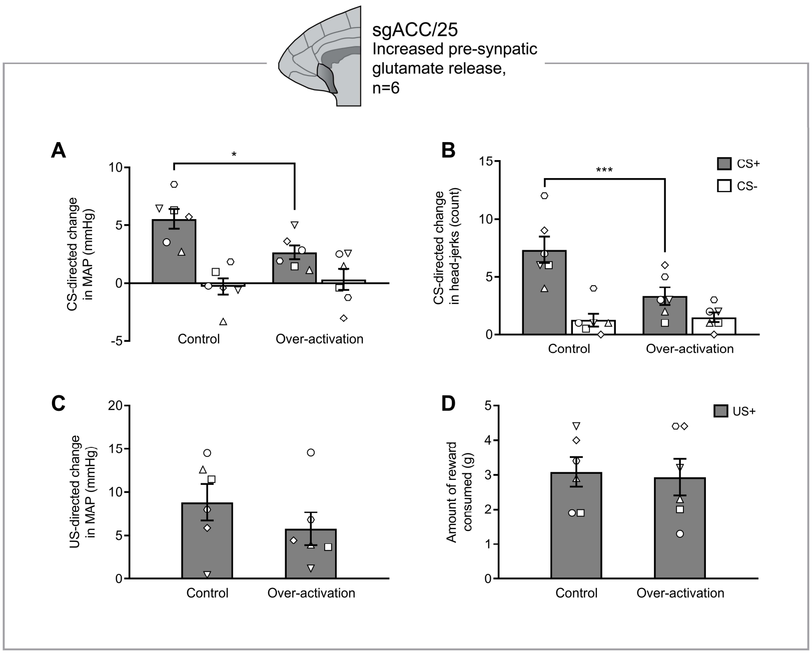

Figure S3. Effects of over-activation of sgACC/25 using CGP/LY. Related to Figure 2. Relevant graphs show mean ± SEM (n=6). **A** sgACC/25 over-activation by increasing pre-synaptic glutamate release (CGP/LY) blunted anticipatory cardiovascular arousal in a CS-dependent manner (manipulation × CS, $F_{1,5}$=14.39, p=0.013) decreasing responding to the CS+ but not the CS- (effect of manipulation: CS+, p=0.014; CS-, p=0.634). **B** The same manipulation blunted anticipatory behavioral arousal in a CS-dependent manner (manipulation × CS, $F_{1,5}$=48.08, p=0.001) decreasing responding to the CS+ but not the CS- (effect of manipulation: CS+, p<0.001; CS-, p=0.839). **C** There was no significant effect on consummatory cardiovascular arousal during the US+ (two-tailed paired *t*-test, p=0.129). **D** There was no significant effect on reward consumption during the US+ (two-tailed paired *t*-test, p=0.665).

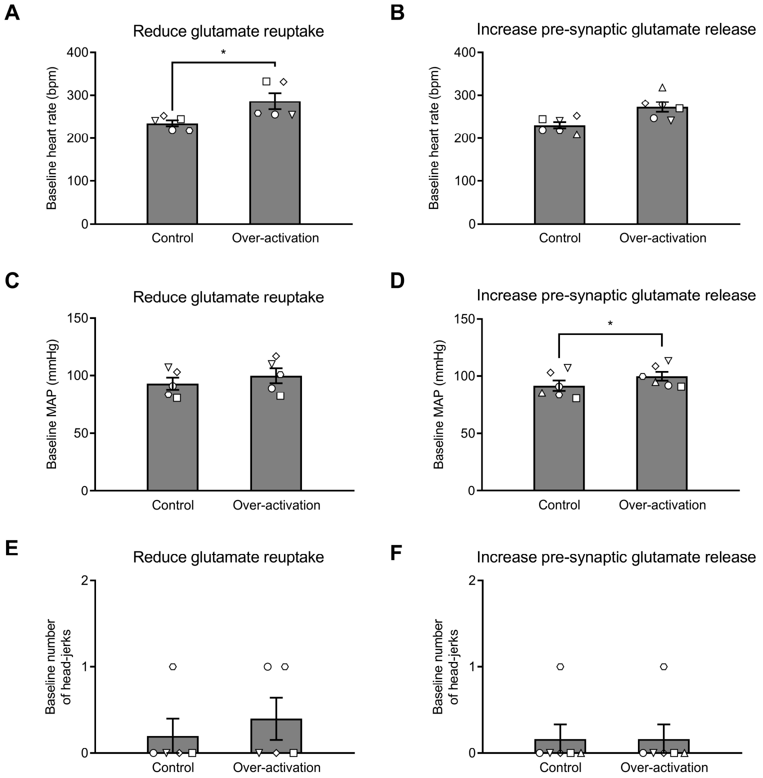

Figure S4. Baseline (20s pre-CS) period effects of sgACC/25 over-activation on HR and MAP. Related to Figures 2 and S3. Relevant graphs show mean ± SEM (n=5 for reduced glutamate reuptake; n=6 for increased pre-synaptic glutamate release). **A** Over-activation of sgACC/25 by reducing glutamate reuptake (DHK) increased baseline HR (two-tailed paired *t*-test, p=0.029). **B** Over-activation of sgACC/25 by increasing pre-synaptic glutamate release (CGP/LY) tended to increase baseline HR (two-tailed paired *t*-test, p=0.051). **C** Reducing glutamate reuptake had no significant effect on baseline MAP (two-tailed paired *t*-test, p=0.097). **D** Increasing pre-synaptic glutamate release increased baseline MAP (two-tailed paired *t*-test, p=0.014). **E** Reducing glutamate reuptake had no significant effect on baseline head-jerk numbers (two-tailed paired *t*-test, p=0.374). **F** Increasing pre-synaptic glutamate release had no effect on baseline head-jerk numbers (values identical across conditions).

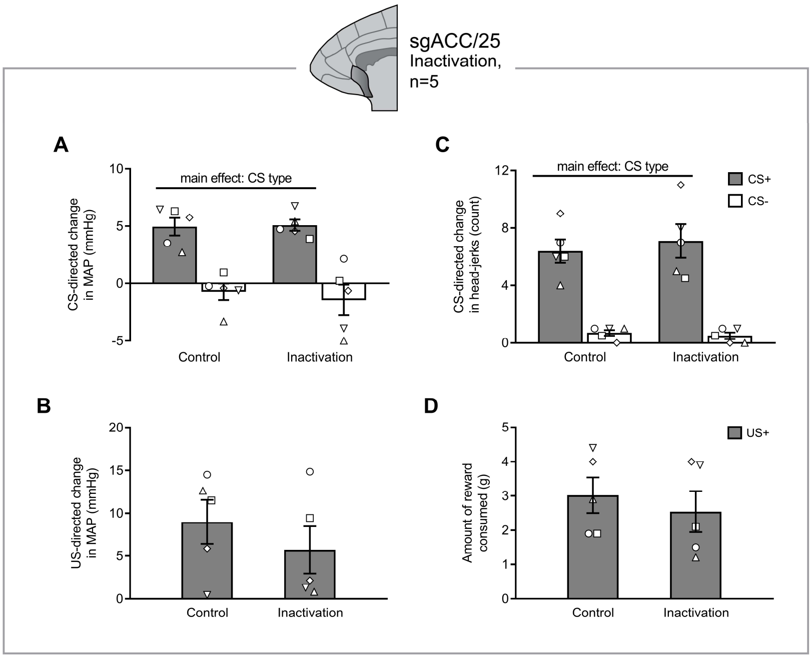

Figure S5. sgACC/25 inactivation had no effect on appetitive anticipatory or consummatory arousal. Related to Figure 2. Relevant graphs show mean ± SEM (n=5). **A** sgACC/25 inactivation using $GABA_A$/$GABA_B$ receptor agonists (muscimol/baclofen, MB) had no effect on anticipatory cardiovascular arousal (manipulation × CS, $F<1$, NS; main effect of CS, $F_{1,4}=31.76$, p=0.005). **B** The same manipulation had no effect on anticipatory behavioral arousal (manipulation × CS, $F_{1,4}=1.59$, p=0.276; main effect of CS, $F_{1,4}=35.27$, p=0.004). **C** There was no significant effect on consummatory cardiovascular arousal during the US+ (two-tailed paired *t*-test, p=0.226). **D** There was no significant effect on reward consumption during the US+ (two-tailed paired *t*-test, p=0.220).

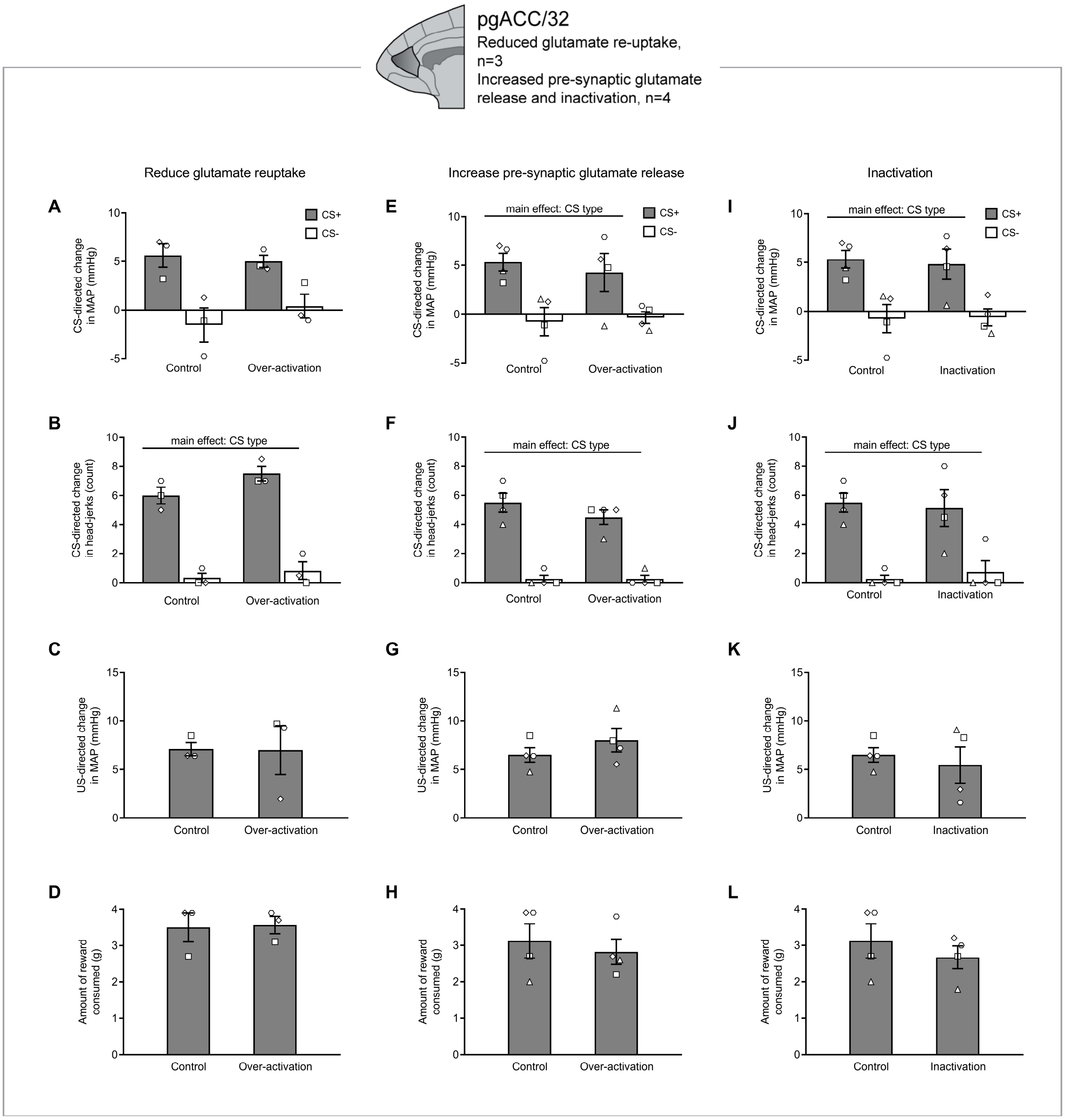

Figure S6. Neither pgACC/32 over-activation nor pgACC/32 inactivation impairs anticipatory or consummatory arousal. Related to Figure 2. Relevant graphs show mean ± SEM (n=4 for inactivation and increased pre-synaptic release; n=3 for reduced glutamate re-uptake). **A** PgACC/32 over-activation by reducing glutamate reuptake (DHK) had no effect on anticipatory cardiovascular arousal (manipulation × CS, $F_{1,2}$=7.77, p=0.108; main effect of CS, $F_{1,2}$=10.07, p=0.087). **B** Reducing glutamate reuptake had no effect on anticipatory behavioral arousal (manipulation × CS, F<1, NS; main effect of CS, $F_{1,2}$=342.3, p=0.003). **C** Reducing glutamate reuptake had no effect on consummatory cardiovascular arousal during the US+ (two-tailed paired t-test, p=0.966). **D** Reducing glutamate reuptake had no effect on reward consumption during the US+ (two-tailed paired t-test, p=0.742). **E** PgACC/32 over-activation by increasing pre-synaptic glutamate release (CGP/LY) had no effect on anticipatory cardiovascular arousal (manipulation × CS, $F_{1,3}$=1.55, p=0.301; main effect of CS, $F_{1,3}$=11.45, p=0.043). **F** Increasing pre-synaptic glutamate release had no effect on anticipatory behavioral arousal (manipulation × CS, $F_{1,3}$=6.00, p=0.092; main effect of CS, $F_{1,3}$=63.71, p=0.004). **G** Increasing pre-synaptic glutamate release had no effect on consummatory cardiovascular arousal

during the US+ (two-tailed paired *t*-test, p=0.450). **H** Increasing pre-synaptic glutamate release had no effect on reward consumption during the US+ (two-tailed paired *t*-test, p=0.484). **I** PgACC/32 inactivation using $GABA_A$/$GABA_B$ receptor agonists (MB) had no effect on anticipatory cardiovascular arousal (manipulation × CS, $F<1$, NS; main effect of CS, $F_{1,3}$=16.50, p=0.027). **J** Inactivation had no effect on anticipatory behavioral arousal (manipulation × CS, $F_{1,3}$=1.77, p=0.275; main effect of CS, $F_{1,3}$=62.85, p=0.004). **K** Inactivation had no effect on consummatory cardiovascular arousal during the US+ (two-tailed paired *t*-test, p=0.646). **L** Inactivation had no significant effect on reward consumption during the US+ (two-tailed paired *t*-test, p=0.122).

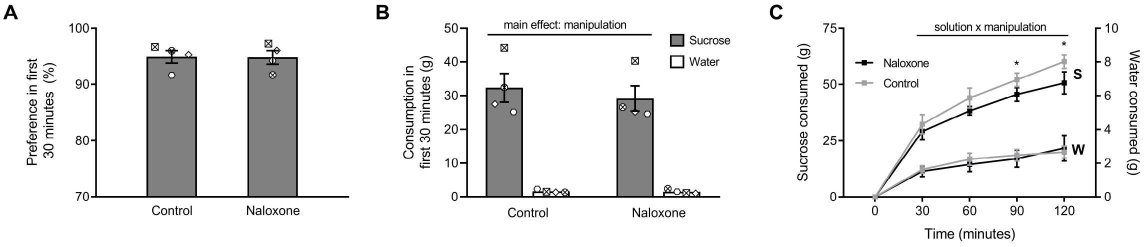

Figure S7. Effects of naloxone, an opioid receptor antagonist, on performance in the sucrose preference test. Related to Figure 3. Relevant graphs show mean ± SEM (n=4). S, sucrose; W, water. **A** Compared to a control injection of saline, the opioid antagonist naloxone had no effect on sucrose preference in the first 30 minutes of the session (two-tailed paired *t*-test, p=0.952). **B** Naloxone reduced both water and sucrose consumption in the first 30 minutes of the session (solution × manipulation, $F_{1,3}$=4.25, p=0.131; main effect of manipulation, $F_{1,3}$=18.00, p=0.024). **C** Across the two-hour session, naloxone reduced cumulative sucrose consumption but not cumulative water consumption (solution × manipulation, $F_{0.532,1.597}$=30.47, p=0.046). Planned comparisons conducted on sucrose and water measurements at each timepoint using Fisher's LSD test revealed a significant decrease in sucrose consumption following naloxone treatment at 90 minutes (p=0.010) and 120 minutes (p=0.024), with no significant effect on water consumption at any timepoint.

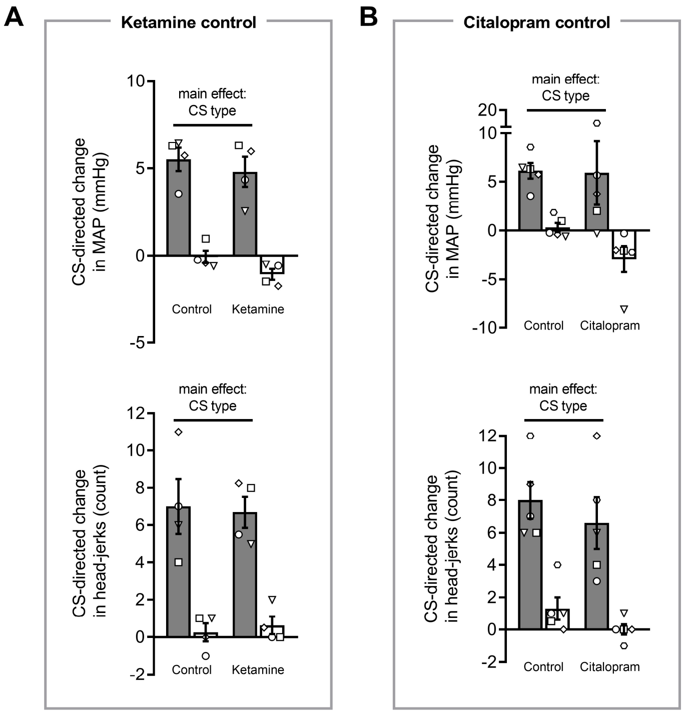

Figure S8. Ketamine and citalopram control experiments (with no intracerebral infusions). Related to Figure 6. Relevant graphs show mean ± SEM (n=4 for ketamine control, n=5 for citalopram control). **A** Ketamine alone (CS-/CS+ sessions in between DHK timepoints) had no effect on cardiovascular (manipulation × CS, F<1, NS; main effect of CS maintained, $F_{1,3}$=86.50, p=0.003) or behavioral (manipulation × CS, F<1, NS; main effect of CS maintained, $F_{1,3}$=31.69, p=0.011) arousal. **B** Citalopram alone had no effect on cardiovascular (manipulation × CS, $F_{1,4}$=1.171, p=0.340; main effect of CS maintained, $F_{1,4}$=19.39, p=0.012) or behavioral (manipulation × CS, $F_{1,4}$<1; main effect of CS maintained, $F_{1,4}$=30.29, p=0.005) arousal.

Tables

Subject	Symbol	Cannulation target	Experimental history	NR. OF INFUSIONS (total)	
				sgACC/25	pgACC/32
Subject 1	□	sgACC/25, pgACC/32	-	16 (21)	5 (9)
Subject 2[i]	△	sgACC/25, pgACC/32	-	3 (3)	3 (3)
Subject 3	▽	sgACC/25	-	13 (16)	n/a
Subject 4	o	sgACC/25	-	10 (15)	n/a
Subject 5	◇	sgACC/25, pgACC/32	Aversive Pavlovian discrimination[ii]	17 (18)	4 (4)
Subject 6	⬡	sgACC/25, pgACC/32	Aversive Pavlovian discrimination[ii]	7 (11)	4 (6)
Subject 7[iii]	◑	sgACC/25, pgACC/32	-	3 (7)	0 (3)
Subject 8[iv]	⊠	sgACC/25, pgACC/32	Resting-state, aversive Pavlovian discrimination[ii]	4 (9)	0 (6)
Subject 9[iv]	⊗	sgACC/25, pgACC/32	Resting-state, aversive Pavlovian discrimination[ii]	6 (11)	0 (4)[v]
Subject 10[vi]	◭	sgACC/25	-	2 (2)	n/a
Subject 11[vi]	⧨	sgACC/25	-	2 (2)	n/a

Table S1. Experimental histories and number of infusions per site. Related to Table 1. The number of infusions (reported far right) includes the number of infusions contributing to the experiments reported here, together with the total number of infusions in brackets (including infusions for piloting DHK doses and infusions for experiments not reported here, carried out after the completion of experiments reported in this manuscript). [i]Due to a problem with implant cement, *Subject 2* lost her cannula implant during the early stages of the infusion protocol on the appetitive Pavlovian discrimination paradigm and was dropped from the study. [ii]*Subjects 5, 6, 8* and *9* had varying amounts of aversive Pavlovian discrimination training prior to the study. This involved 30-minute sessions five days a week, in which marmosets were presented with auditory cues which were paired either with a mildly aversive loud noise (0.3-0.7s, 115-118dB) or a neutral event (0.5s house-light off). *Subjects 5* and *6* failed to learn the discrimination and so testing was terminated and they were then transferred to the appetitive paradigm. *Subjects 8* and *9* additionally received infusions in an emotionally neutral resting-state condition prior to the study. [iii]*Subject 7* had telemetric probe failure early on during appetitive Pavlovian discrimination and so was moved onto PET imaging, for which cardiovascular measurements were not essential. [iv]*Subjects 8* and *9* had telemetry probe failures and so for these subjects, testing was conducted on appetitive paradigms which did not require cardiovascular measurements. [v]Unilateral cannula placement. [vi]*Subjects 10* and *11* took part in other studies following their human intruder test, the results of which are not reported here.

Infusion	Latency (seconds, mean ± SEM)	P value
sgACC/25 control	14.44 ± 2.35	
sgACC/25 DHK	23.36 ± 8.91	0.252
sgACC/25 CGP/LY	12.91 ± 2.95	0.485
sgACC/25 MB	20.13 ± 7.28	0.609
pgACC/32 control	15.05 ± 4.60	
pgACC/32 DHK	6.03 ± 1.49	0.244
pgACC/32 CGP/LY	13.75 ± 5.50	0.8
pgACC/32 MB	20.13 ± 7.28	0.776

Table S2. Consummatory (US+) latencies to start eating food reward. Related to Figures 2, S3, S5 and S6. P values reported from two-tailed paired *t*-tests comparing control (saline) sessions *vs.* drug sessions.

Locomotion values (s)	Subject						Mean ± SEM
	○	□	◇	▽	⬡	△	
Baseline							
Control	0.00	0.00	0.00	0.97	0.27	0.00	0.21 ± 0.16
sgACC/25 DHK	0.56	0.80	0.90	0.00	0.96	-	0.64 ± 0.17
sgACC/25 CGP/LY	0.00	0.00	0.00	0.00	1.92	1.73	0.61 ± 0.39
CS							
Control	1.33	1.90	0.35	0.62	1.23	1.04	1.08 ± 0.22
sgACC/25 DHK	1.34	0.93	1.91	0.71	1.21	-	1.22 ± 0.20
sgACC/25 CGP/LY	0.90	1.59	0.81	0.46	1.04	0.00	0.80 ± 0.22

Table S3. Assessment of locomotor activity during sgACC/25 control and over-activation infusion sessions. Related to Figures 2 and S3. Over-activation using DHK did not change baseline or CS locomotion (manipulation × phase, F<1, NS; main effect of manipulation, F<1, NS; main effect of phase, $F_{1,4}$=19.83, p=0.011). Over-activation using CGP/LY release did not change baseline or CS locomotion (manipulation × phase, $F_{1,5}$=1.47, p=0.279; main effect of manipulation, F<1, NS; main effect of phase, $F_{1,5}$=2.78, p=0.157). We additionally correlated CS-directed changes in MAP and CS-directed changes in locomotion across control, DHK and CGP/LY infusion types and found no evidence for a correlation (control, DHK and CGP/LY, R^2=0.052; not shown) supporting the notion that CS-directed changes in MAP are unrelated to alterations in locomotion.

Source of variation		ANOVAs on absolute MAP values compared to control						ANOVAs on head-jerk values compared to control					
		sgACC/25 DHK	sgACC/25 CGP/LY	sgACC/25 MB	pgACC/32 DHK	pgACC/32 CGP/LY	pgACC/32 MB	sgACC/25 DHK	sgACC/25 CGP/LY	sgACC/25 MB	pgACC/32 DHK	pgACC/32 CGP/LY	pgACC/32 MB
Manipulation (control *vs.* drug)		0.225	0.011	0.840	0.957	0.127	0.152	0.013	0.003	0.044	0.744	0.444	0.604
Phase (baseline *vs.* CS+)		<0.001	<0.001	<0.001	0.020	0.032	0.020	0.002	0.002	0.002	0.004	0.004	0.015
Manipulation × Phase interaction		0.045	0.029	0.900	0.660	0.575	0.704	0.006	0.003	0.128	0.633	0.444	>0.999
Post-hoc (baseline *vs.* CS+)	Control	0.003	0.003	N/A				<0.001	<0.001	N/A			
	Drug	0.353	0.014					0.073	0.002				

Table S4. Analysis of variance (ANOVA) conducted on absolute MAP values and head-jerk values during baseline and CS+ periods for all appetitive Pavlovian infusion results. Related to Figures 2, S3, S5 and S6. Highlighted in grey are significant results at a threshold of $\alpha=0.05$. **MAP:** All comparisons showed a main effect of phase (indicating MAP values were significantly different between baseline and CS+ periods). Only sgACC/25 DHK and sgACC/25 CGP/LY infusions showed a manipulation × phase interaction, evidencing a differential effect on the baseline *vs.* CS+ period compared to control infusions. Post-hoc comparisons using Sidak's multiple comparisons test revealed that CS+ period absolute MAP did not significantly differ from baseline period values in the case of sgACC/25 DHK, indicating an abolition of appetitive anticipatory arousal. Whilst CS+ period absolute MAP values were still significantly different from baseline absolute MAP values in the case of sgACC/25 CGP/LY infusions, the magnitude of the difference was less than control infusions (control: 5.56 ± 0.86, CGP/LY: 2.66 ± 0.60, mean ± SEM; see **Fig. S3A**) consistent with a reduction but not abolition of anticipatory cardiovascular arousal. **Head-jerks:** All comparisons showed a main effect of phase (indicating head-jerk values were significantly different between baseline and CS+ periods). Only sgACC/25 DHK and sgACC/25 CGP/LY infusions showed a manipulation × phase interaction, evidencing a differential effect on the baseline *vs.* CS+ period compared to control infusions. Post-hoc comparisons using Sidak's multiple comparisons test revealed that CS+ period head-jerks did not significantly differ from baseline period values in the case of sgACC/25 DHK, indicating an abolition of appetitive anticipatory behavioral arousal. Whilst CS+ period head-jerk values were still significantly different from baseline head-jerk values in the case of sgACC/25 CGP/LY infusions, the magnitude of the difference was less than control infusions (control: 7.39 ± 0.50, CGP/LY: 3.50 ± 0.50, mean ± SEM; see **Fig. S3B**) consistent with a reduction but not abolition of anticipatory behavioral arousal.

Extracted factors	Percentage of variance explained	Behavior	Loading onto factor 1, representing anxiety
**1**	39.7317	Average height	0.816
2	14.3895	Bobs	0.769
3	10.2584	TSAB	0.688
4	9.0836	Tse egg calls	0.417
5	8.2543	Egg calls	0.332
6	6.9894	Tsik egg calls	0.323
7	4.0461	Tsik calls	-0.091
8	4.0321	Locomotion	-0.568
9	3.2148	TSAF	-0.790

Table S5. Exploratory factor analysis (EFA) and factor loadings of individual behaviors. Related to Figure 4. TSAB, time spent at back. TSAF, time spent at front. An EFA was carried out on the behavioral data of 171 marmosets undergoing the human intruder test as part of a screening protocol. Based on the point of inflection of a scree plot, a single factor (1) was identified accounting for 39.7% of variance. The pattern in which the individual behaviors load onto this factor suggest that it represents the marmosets' anxiety towards the intruder: a high factor score represents an animal high up and at the back of cage, far away from the intruder, remaining relatively still and performing a lot of head bobs, indicative of high anxiety.

Phase	Subject	Manipulation	TSAF, %	TSAB, %	Height, cm	Locm., %	Bobs	Tsik	Tsik-egg	Tse	Tse-egg	Egg
**Baseline**	◭	Control	23.48	0.00	24.13	4.75						
		Over-activation	14.70	0.00	25.67	7.09						
	⧩	Control	52.99	0.00	57.35	4.98						
		Over-activation	84.82	0.00	49.14	3.77						
	⊗	Control	91.95	2.23	29.77	8.34						
		Over-activation	72.96	12.81	37.71	7.48						
	Summary	Control mean	56.14	0.74	37.08	6.02						
		Control SEM	19.83	0.74	10.26	1.16						
		Over-activation mean	57.49	4.27	37.51	6.11						
		Over-activation SEM	21.67	4.27	6.78	1.18						
**Intruder**	◭	Control	61.94	9.82	45.35	8.6	4	0	0	0	0	0
		Over-activation	4.00	41.38	50.17	8.23	30	1	10	3	13	0
	⧩	Control	42.49	0.00	27.52	1.76	1	0	0	0	0	0
		Over-activation	8.02	31.02	68.73	4.51	1	4	3	0	0	0
	⊗	Control	38.29	0.86	43.64	9.03	10	0	1	0	4	2
		Over-activation	22.27	24.98	51.08	7.29	21	7	16	5	1	4
	Summary	Control mean	47.57	3.56	38.84	6.46	5	0	0	0	1	1
		Control SEM	7.28	3.14	5.68	2.35	3	0	0	0	1	1
		Over-activation mean	11.43	32.46	56.66	6.68	17	4	10	3	5	1
		Over-activation SEM	5.54	4.79	6.04	1.12	9	2	4	1	4	1

Table S6. **Individual behavioral measures during the two-minutes prior to intruder exposure (baseline) and two-minute intruder period (intruder) across control and over-activation conditions. Related to Figure 4.** TSAF, time spent at front. TSAB, time spent at back. Locm., locomotion. Marmosets do not exhibit any bobbing or tsik, tsik-egg, tse, tse-egg or egg vocalization behaviors during the baseline period, so these were not scored.

Manipulation	Hemisphere	sgACC/25 SUVR values by subject				Mean ± SEM
		□	▽	◇	◑	
Control	Left	0.856	1.028	0.843	0.822	0.887 ± 0.047
	Right	0.785	1.002	0.842	0.833	0.866 ± 0.047
Over-activation	Left	1.069	1.157	0.921	1.019	1.041 ± 0.049
	Right	1.023	1.040	0.933	0.992	0.997 ± 0.023
Over-activation + Ketamine	Left	1.017	0.974	0.882	0.807	0.920 ± 0.047
	Right	0.967	0.905	0.880	0.807	0.890 ± 0.033

Table S7. Measurements of SUVR changes across control, over-activation and [over-activation + ketamine] in an atlas-defined sgACC/25 ROI. Related to Figure 7. Within this ROI, there was a significant effect of manipulation on SUVR values (manipulation × hemisphere, $F_{2,6}$<1, NS; effect of manipulation: $F_{2,6}$=6.22, p=0.034). Planned comparisons using Fisher's LSD test revealed a significant difference between control *vs.* over-activation (p=0.016) and over-activation *vs.* [over-activation + ketamine] (p=0.037) conditions, but not for control *vs.* [over-activation + ketamine] (p=0.530) conditions.

**Drug or drug cocktail**	**Mechanism**	**Route**	**Dose**	**Rate**	**Pre-treatment**
Dihydrokainic acid (DHK)	EAAT2 antagonist	Central infusion	1.35µg/µL	0.5µL/min	10 minutes
CGP52432/LY341495 (CGP-LY)	$GABA_B$/$mGlu_{2/3}$ receptor antagonist	Central infusion	10ng/µL CGP52432 100pg/µL LY341495	0.5µL/min	15 minutes
Muscimol/Baclofen (MB)	$GABA_A$/$GABA_B$ receptor agonist	Central infusion	11.4ng/µL muscimol 0.214µg/µL baclofen	0.25µL/min	25 minutes
Ketamine	NMDA receptor antagonist	Intramuscular injection	0.5mg/kg	n/a	n/a
Citalopram	SSRI	Intramuscular injection	10mg/kg	n/a	30 minutes
Naloxone	Non-selective opioid receptor antagonist	Intramuscular injection	10mg/kg	n/a	10 minutes

Table S8. Mechanism, route of administration, dose and pre-treatment time for drugs used in the study. Related to Methods: *Drug treatments*. Pre-treatment refers to the time interval between completion of infusion and entry of the animal into the behavioral testing apparatus. All centrally administered drugs were infused over two minutes and injectors were left in place for one minute to facilitate adequate diffusion.
