# Supplementary material for: Fractionating Blunted Reward Processing Characteristic of Anhedonia by Over-Activating Primate Subgenual Anterior Cingulate Cortex
Source: Neuron. 2019 Jan 16;101(2):307–320.e6. doi: 10.1016/j.neuron.2018.11.021 (PMC6344231; doi:10.1016/j.neuron.2018.11.021)
Supplement: Document S2. Article plus Supplemental Information [file mmc2.pdf]

Neuron
Article

Fractionating Blunted Reward Processing Characteristic of Anhedonia by Over-Activating Primate Subgenual Anterior Cingulate Cortex

Highlights

- Primate sgACC/25 over-activation blunts reward anticipation, but not consumption
- Over-activation of sgACC/25 also blunts willingness to work for reward
- Anticipatory blunting involves metabolic changes in reward-related circuits
- Acute ketamine reverses blunted anticipatory arousal and metabolic changes

Authors

Laith Alexander, Philip L.R. Gaskin, Stephen J. Sawiak, ..., Gemma J. Cockcroft, Hannah F. Clarke, Angela C. Roberts

Correspondence

acr4@cam.ac.uk

In Brief

Using intracerebral microinfusions in marmosets, Alexander et al. demonstrate a causal role for sgACC/25 over-activity in specific aspects of impaired reward processing associated with anhedonia. Ketamine successfully ameliorates related impairments by modulating activity within a dysfunctional neural circuit involved in reward processing and interoception.

Alexander et al., 2019, Neuron *101*, 307–320
January 16, 2019 © 2018 The Authors. Published by Elsevier Inc.
https://doi.org/10.1016/j.neuron.2018.11.021

CellPress

Fractionating Blunted Reward Processing Characteristic of Anhedonia by Over-Activating Primate Subgenual Anterior Cingulate Cortex

Laith Alexander,[1,2] Philip L.R. Gaskin,[1,2,4] Stephen J. Sawiak,[2,3] Tim D. Fryer,[3] Young T. Hong,[3] Gemma J. Cockcroft,[1,2] Hannah F. Clarke,[1,2] and Angela C. Roberts[1,2,5,*]
[1]Department of Physiology, Development and Neuroscience, University of Cambridge, Cambridge CB2 3DY, UK
[2]Behavioural and Clinical Neuroscience Institute, University of Cambridge, Cambridge CB2 3EB, UK
[3]Wolfson Brain Imaging Centre, Department of Clinical Neurosciences, University of Cambridge, Cambridge CB2 0QQ, UK
[4]Present address: BenevolentAI, London, UK
[5]Lead Contact
*Correspondence: acr4@cam.ac.uk
https://doi.org/10.1016/j.neuron.2018.11.021

SUMMARY

Anhedonia is a core symptom of depression, but the underlying neurobiological mechanisms are unknown. Correlative neuroimaging studies implicate dysfunction within ventromedial prefrontal cortex, but the causal roles of specific subregions remain unidentified. We addressed these issues by combining intracerebral microinfusions with cardiovascular and behavioral monitoring in marmoset monkeys to show that over-activation of primate subgenual anterior cingulate cortex (sgACC, area 25) blunts appetitive anticipatory, but not consummatory, arousal, whereas manipulations of adjacent perigenual ACC (pgACC, area 32) have no effect. sgACC/25 over-activation also reduces the willingness to work for reward. ^{18}F-FDG PET imaging reveals over-activation induced metabolic changes in circuits involved in reward processing and interoception. Ketamine treatment ameliorates the blunted anticipatory arousal and reverses associated metabolic changes. These results demonstrate a causal role for primate sgACC/25 over-activity in selective aspects of impaired reward processing translationally relevant to anhedonia, and ketamine's modulation of an affective network to exert its action.

INTRODUCTION

Major depressive disorder (MDD) is a common and debilitating condition that contributes significantly to global disease burden (Ferrari et al., 2013). Anhedonia—defined as a loss of interest or pleasure in all or almost all activities—is a core feature of MDD as outlined by the Diagnostic and Statistical Manual of Mental Disorders (DSM-V; American Psychiatric Association, 2013). The clinical importance of anhedonia is illustrated by its high prevalence (Keller et al., 1995) and its robustness as a negative prognostic indicator (Spijker et al., 2001; Uher et al., 2012). Despite this, anhedonia remains poorly characterized both psychologically and neurobiologically.

Psychologically, the majority of studies fail to recognize its distinct behavioral subtypes, including anticipatory, motivational, and consummatory components (for discussion, see Der-Avakian and Markou, 2012; Treadway and Zald, 2011). Instead, clinical and preclinical measures of anhedonia are almost exclusively consummatory. Clinical studies use scales to measure anhedonia such as the Fawcett-Clark Pleasure Scale (Fawcett et al., 1983) and the Chapman Physical Anhedonia Scale (Chapman et al., 1976), in which the items are primarily concerned with the hedonic (consummatory) responses to reward. Similarly, rodent studies typically measure sucrose consumption/preference as an overall index of anhedonia (Ferenczi et al., 2016; Tye et al., 2013). However, there is a fundamental disconnect between the construct assessed in these studies and the pattern of impairments manifested in people with depression, who typically display anhedonic symptoms in anticipatory and motivational domains (Klein, 1987; McFarland and Klein, 2009; Treadway and Zald, 2011) with relatively intact consummatory responses to rewards (Amsterdam et al., 1987; Arrondo et al., 2015; Berlin et al., 1998; Dichter et al., 2010; although see McCabe et al., 2012 for neural changes during consummatory processing in at-risk groups).

Neurobiologically, while correlative human neuroimaging studies have implicated subregions of the ventromedial prefrontal cortex (vmPFC) in the etiology of depression, the precise anatomical locus of these changes varies throughout the subgenual anterior cingulate cortex (sgACC) and the perigenual anterior cingulate cortex (pgACC). In depressed subjects, over-activity in sgACC (including area 25) has been reported (Drevets et al., 2008; Keedwell et al., 2009; Mayberg et al., 2005), together with increased resting-state functional connectivity of this region to the default-mode network (Greicius et al., 2007). In neighboring pgACC (including area 32), there are variable reports of under-activity (Fitzgerald et al., 2008; Ito et al., 1996; Mayberg et al., 1994) and over-activity (Drevets et al., 1992; Ebert and Ebmeier, 1996). Comparatively fewer studies have assessed the involvement of these regions in anhedonia specifically and

This is an open access article under the CC BY license (http://creativecommons.org/licenses/by/4.0/).

Table 1. Summary of Subjects that Took Part in the Study

			Fractionating Anhedonia			Responsivity to Treatment		Circuitry	
			Appetitive Pavlovian Discrimination	Progressive Ratio	Sucrose Preference	Ketamine	Citalopram	PET Scanning	Human Intruder Test
Subject	Symbol	Cannulation Target	n = 6	n = 3	n = 4	n = 4	n = 5	n = 4	n = 3
Subject 1	□	sgACC/25, pgACC/32	✓ *a*			✓ *b*	✓ *c*	✓ *d*	
Subject 2	△	sgACC/25, pgACC/32	✓ *a*						
Subject 3	▽	sgACC/25	✓ *a*			✓ *c*	✓ *b*	✓ *d*	
Subject 4	○	sgACC/25	✓ *a*			✓ *b*	✓ *c*		
Subject 5	◇	sgACC/25, pgACC/32	✓ *a*	✓ *f*	✓ *e*	✓ *b*	✓ *c*	✓ *d*	
Subject 6	⬡	sgACC/25, pgACC/32	✓ *a*		✓ *c*		✓ *b*		
Subject 7	◑	sgACC/25, pgACC/32						✓ *a*	
Subject 8	⊠	sgACC/25, pgACC/32		✓ *b*	✓ *a*				
Subject 9	⊗	sgACC/25, pgACC/32		✓ *c*	✓ *a*				✓ *b*
Subject 10	▲	sgACC/25							✓ *a*
Subject 11	▼	sgACC/25							✓ *a*

A tick indicates the subject took part in that phase of the study. *a–f* depict the order in which each subject undertook the various phases.

those that have implicate pgACC/32 (Harvey et al., 2007; Keedwell et al., 2005) and more caudal sgACC (Dunn et al., 2002), but their causal roles remain unknown. Although vmPFC lesions in humans have been linked to reduced anticipatory arousal prior to risky decisions (Bechara et al., 1996), the interpretation of these results is confounded by a lack of anatomical specificity—the damage associated with lesions is neither restricted to a specific vmPFC subregion, nor is it restricted to gray matter (damaging underlying fibers of passage). Similarly, the only non-human primate study investigating the role of sgACC/25 in reward-related behavior utilized aspiration lesions, complicating interpretation due to white matter damage (Rudebeck et al., 2014).

While targeted interventional studies in rodents have also attempted to address the issue of causality, progress has been hampered and translation made difficult owing to (1) a lack of understanding regarding the extent to which anatomical homology relates to functional equivalence (Myers-Schulz and Koenigs, 2012; Wallis et al., 2017), (2) a failure to differentiate between the functionally distinct prelimbic (PL) and infralimbic (IL) mPFC sectors in the rodent (for example, Ferenczi et al., 2016), and (3) a lack of validity of the rodent sucrose consumption test as a measure of anhedonia relevant to depression (Dwyer, 2012). The issue is therefore best addressed by using interventional studies in non-human primates in which the anatomical organization of the vmPFC most closely resembles that of humans, and by recognizing the distinct subtypes of reward-related deficits that likely underlie anhedonia.

The present study determined whether over-activity in sgACC/25 and under-/over-activity in pgACC/32 reported in depressed humans can cause translationally relevant deficits in reward processing in a non-human primate. By combining intracerebral infusions with positron emission tomography (PET) imaging of the glucose analog 2-deoxy-2-[^{18}F]fluoro-D-glucose (^{18}F-FDG), we also determined the network of brain regions associated with the observed deficits. Since ketamine has recently emerged as a promising glutamate-based antidepressant demonstrating efficacy in treating reward processing deficits (Lally et al., 2014, 2015; Parsaik et al., 2015), we determined the efficacy of ketamine to alleviate these deficits along with associated circuit-wide changes.

RESULTS

An overview of the subjects used in these experiments is shown in Table 1. Additional details of experimental histories together with numbers of infusions are shown in Table S1. Histological assessment of cannula placement and drug-induced *c-fos* expression is shown in Figure S1.

sgACC/25 Over-Activation Blunts Anticipatory, but Not Consummatory, Arousal for Reward, whereas pgACC/32 Manipulations Have No Effect

Following surgery and recovery (experimental outline shown in Figure 1A), marmosets (n = 6) acquired appetitive Pavlovian discriminative cardiovascular and behavioral arousal responses to an auditory conditioned stimulus (CS+) predicting the presence of high-incentive food reward (unconditioned stimulus, US+), but not to a second auditory stimulus (CS−) predicting the absence of food reward (US−; Figures 1B and 1C). Successful discrimination was evident in cardiovascular responses as an increase in mean arterial blood pressure (MAP) during the CS+ (compared to the 20 s pre-CS baseline period), but not during the CS− (Figures 1C and S2A). During the US+ consummatory period, a rise in MAP was observed above the rise seen during the CS+ with no change during the US− (Figures 1C and S2B). Marmosets fail to show MAP rises when consuming non-preferred foods (Braesicke et al., 2005), suggesting that the increase observed during the US+ period was due to hedonic—rather than ingestive—factors. Heart rate (HR) responses were variable: while there was a trend toward discrimination during the CS period (Figure S2C), no discrimination was evident during

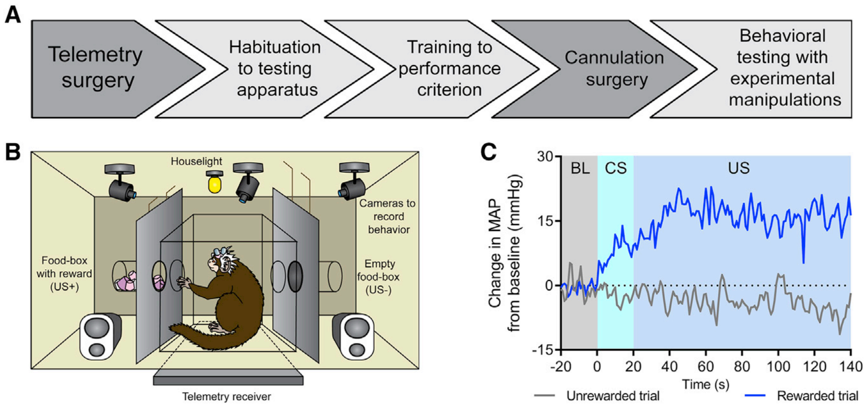

Figure 1. Experimental Outline and Conditioned Discrimination

Mean arterial pressure (MAP) values are recorded in mmHg.

(A) Experimental overview. Following telemetry surgery, marmosets were habituated to the testing apparatus for 5–10 sessions, trained on the appetitive discrimination task until criterion was reached (significant MAP discrimination over three CS+/CS− sessions, two-tailed paired t test), and then cannulated to target sgACC/25 alone or both sgACC/25 and pgACC/32. Following re-attainment of criterion post-surgery, experimental manipulations took place.

(B) Diagram of conditioning apparatus. During discrimination sessions, two auditory cues predicted either the presence (CS+/US+) or absence (CS−/US−) of a high-incentive food reward (marshmallow). A telemetry receiver placed underneath the apparatus recorded cardiovascular measurements, which were sent to a computer in an adjacent room.

(C) Example MAP trace during baseline (BL, 20 s immediately prior to CS), CS (20 s), and US (120 s) periods for a rewarded and non-rewarded trial within a conditioning session. Values are calculated as a difference from the mean MAP during baseline. Animals showed an anticipatory MAP rise during the CS+ and a further consummatory rise during the US+.

the US period (Figure S2D). MAP is therefore used as the principal cardiovascular measurement throughout the study owing to its sensitivity as a discriminative measure of anticipatory and consummatory arousal.

Behaviorally, both discriminative conditioned CS-directed and conditioned US-directed behaviors were exhibited during the CS period. The principal CS-directed behavior was a rapid "head-jerk," previously described in rodents (Holland, 1977) and marmosets (Reekie et al., 2008) as an orienting response to an auditory appetitive CS. Animals developed increased head-jerking behavior during the CS+, but not the CS− (Figure S2E). The US-directed measure used was nose-poking toward the feeder box, but this did not discriminate between CS type (Figure S2F). During the US+, the amount of food consumed was used as the behavioral index of reward consumption. The latency to begin eating food reward was also measured.

To over-activate sgACC/25, marmosets received infusions of dihydrokainic acid (DHK; excitatory amino acid transporter-2 [EAAT2] inhibitor) to reduce glutamate reuptake (n = 5) and/or CGP52432/ LY341495 (CGP/LY; $GABA_B$ receptor antagonist/ $mGlu_{2/3}$ receptor antagonist) to increase pre-synaptic glutamate release (n = 6). DHK-induced over-activation of sgACC/25 blunted anticipatory, but not consummatory, arousal, selectively reducing cardiovascular and behavioral responses during the anticipatory CS+ period (Figures 2A and 2B), but not during the consummatory US+ period (Figures 2C and 2D). CGP/LY over-activation of sgACC/25 resulted in the same pattern of blunting (Figure S3). Neither manipulation caused a significant change in the latency to eat the food reward (Table S2), nor were there any changes in locomotor activity during the baseline or CS periods (Table S3). Both methods of over-activation tended to elevate HR during the baseline period (Figures S4A and S4B). DHK infusions had no effect on baseline MAP (Figure S4C), while CGP/LY infusions elevated baseline MAP (Figure S4D). Nevertheless, the effect of CGP/LY to blunt CS+ cardiovascular arousal is unlikely due to a ceiling effect associated with raised baseline MAP because MAP still increased during the consummatory period. Neither method had any effect on baseline head-jerk numbers (which were infrequent; Figures S4E and S4F). In contrast to sgACC/25 over-activation, we found no effect of sgACC/25 inactivation (muscimol/baclofen [MB]; $GABA_A$/$GABA_B$ receptor agonist) on arousal during either reward anticipation or consumption (Figure S5), suggesting that sgACC/25 activity is not necessary for the expression of these appetitive responses.

Despite numerous neuroimaging studies implicating both under- and over-activity in pgACC/32 in depression and anhedonia, we found that neither bilateral pgACC/32 over-activation (using DHK or CGP/LY) nor bilateral pgACC/32 inactivation (using MB) had any effect on anticipatory CS or consummatory US arousal (Figure S6). This suggests that activity changes in pgACC/32 are not causally related to basic reward processing deficits and may reflect deficits in using reward information in decision-making (Amemori et al., 2015) or compensatory changes.

Additional analyses of variance were carried out on absolute MAP values and head-jerk values (rather than CS-directed values) using phase as a factor (baseline versus CS+) for all sgACC/25 and pgACC/32 infusions. These results are reported in Table S4 and confirm the conclusions reported above.

sgACC/25 Over-Activation Impairs the Willingness to Work for Reward on an Instrumental Progressive Ratio Schedule of Reinforcement

To characterize the extent of the reward processing deficit further, the effects of over-activation of sgACC/25 (using DHK) on instrumental progressive ratio performance were assessed. Marmosets (n = 3) were trained to respond to a visual stimulus on a touchscreen under increasingly demanding reinforcement requirements until a breakpoint (2 min of inactivity) was reached (Figures 3A and 3B). Bilateral over-activation of sgACC/25 significantly impaired progressive ratio performance, reducing the breakpoint to levels significantly below both the previous day (−70.3% ± 12.5%; mean ± SEM) and control infusions (−69.5% ± 11.6%; mean ± SEM) (Figure 3C). This effect was

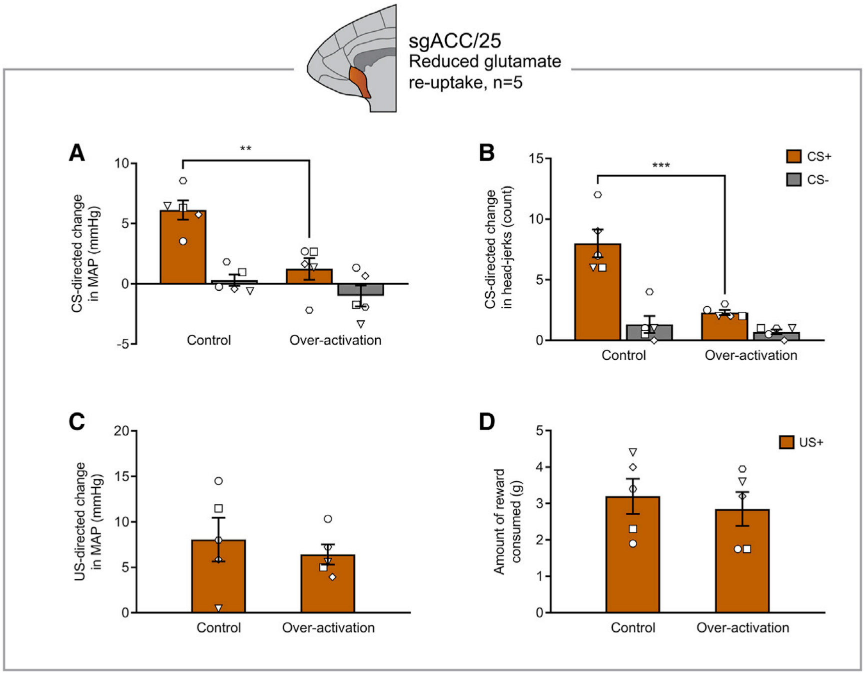

Figure 2. sgACC/25 Over-Activation by Reducing Glutamate Reuptake Blunts Appetitive Anticipatory Arousal, but Not Consummatory Arousal

Relevant graphs show mean ± SEM (n = 5).

(A) sgACC/25 over-activation by reducing glutamate reuptake (DHK) blunted anticipatory cardiovascular arousal in a CS-dependent manner (manipulation × CS, $F_{1,4}$ = 10.63, p = 0.031), decreasing responding to the CS+, but not the CS− (effect of manipulation: CS+, p = 0.006; CS−, p = 0.301).

(B) The same manipulation also blunted anticipatory behavioral arousal in a CS-dependent manner (manipulation × CS, $F_{1,4}$ = 72.25, p = 0.001), decreasing responding to the CS+, but not the CS− (effect of manipulation: CS+, p < 0.001; CS−, p = 0.407).

(C) There was no significant effect on consummatory cardiovascular arousal during the US+ (two-tailed paired t test, p = 0.451).

(D) There was no significant effect on reward consumption during the US+ (two-tailed paired t test, p = 0.241).

independent of individual marmosets' baseline level of responding—higher and lower responders showed similar, marked impairments (Figure 3D).

sgACC/25 Over-Activation Has No Effect on Sucrose Preference or Consumption, Despite These Being Common Preclinical Analogs of Anhedonia

We also investigated reward consumption in a manner directly comparable to rodent studies using a sucrose preference test adapted for marmosets (n = 4; Figure 3E). Measurements of sucrose and water consumption were taken every 30 min across a 2-hr testing session, with an *a priori* interest in the first 30-min window owing to the rapid action of intracranial infusions. In the session prior to manipulations, marmosets showed a high preference for sucrose solution over water and consumed large amounts of sucrose in both the first 30 min and across the 2-hr testing window (Figures 3F and 3G). As a positive control, we assessed the effects of peripheral injections of the opioid antagonist naloxone—a putative modulator of the hedonic "liking" system. In the first 30 min of the session, naloxone had no effect on sucrose preference (Figure S7A) but did reduce both sucrose consumption and water consumption (Figure S7B). Across the entire 2-hr period, naloxone reduced sucrose consumption without affecting water consumption with the strongest effects at later time points (Figure S7C). When assessing potential consummatory effects induced by sgACC/25 over-activation, we therefore measured both sucrose preference (reduced in rodent models of depression) and absolute sucrose consumption (reduced by naloxone and in rodent models of depression) to fully ascertain the presence of any potential consummatory impairment. Over-activation of sgACC/25 had no effect on sucrose preference or consumption in the first 30 min (Figures 3H and 3I), nor across the 2-hr session (Figure 3J), demonstrating that while over-activity in this region can cause anticipatory and motivational impairments, it has no obvious effect on reward consumption.

sgACC/25 Over-Activation Does Not Cause a General Blunting in Emotional Arousal

Despite having no effect on consummatory arousal, it is still possible that the anticipatory and motivational impairments observed following sgACC/25 over-activation are due to a more general blunting of effect. We therefore determined the effects of sgACC/25 over-activation (using DHK) on emotional arousal in the human intruder test (n = 3), where marmosets are confronted with an uncertain threat in the form of a non-familiar human. When confronted with an intruder in their home cage, marmosets maintain distance, show rapid side-to-side "bobbing" movements, and exhibit a range of vocalizations indicative of an anxiety response (Figure 4A). Using an exploratory factor analysis (EFA; STAR Methods), we have loaded these behaviors onto a single latent factor representing marmosets' anxiety toward the human intruder (Table S5). A high factor (anxiety) score typically reflects high distance, high bobbing, and increased vocalizations.

In the 2-min baseline period prior to intruder exposure, there were no differences in behavioral measures between control and over-activation conditions (Table S6). During intruder exposure, sgACC/25 over-activation significantly enhanced responsivity of marmosets to the human intruder reflected in the increased anxiety score (Figure 4B; see Table S6 for individual measures). The effect of sgACC/25 to elevate arousal in response to an intruder is the opposite finding to that expected from a general emotional blunting, suggesting instead that the effects of sgACC/25 over-activation are dependent on the affective context—however, the extent to which this emotional

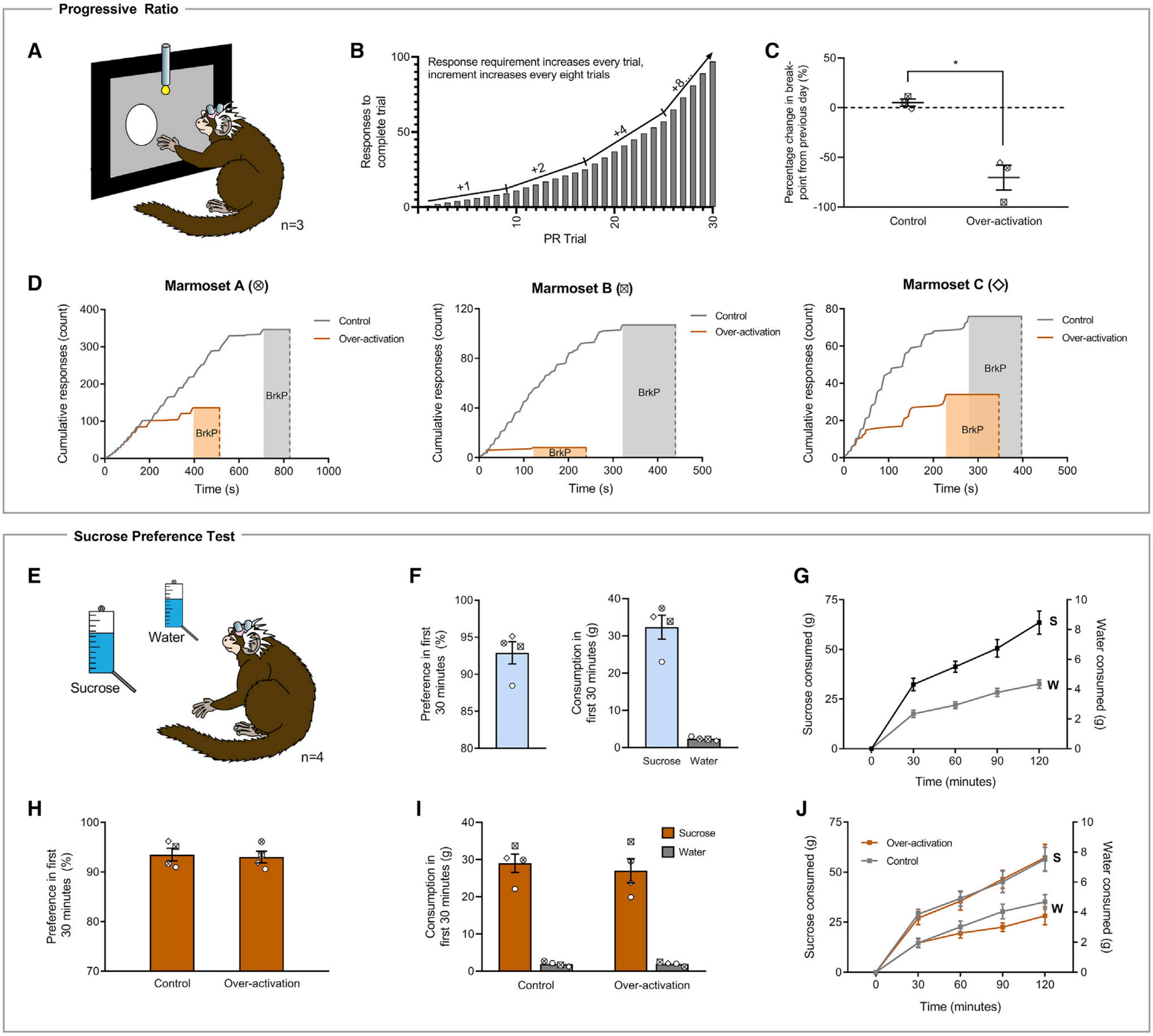

Figure 3. sgACC/25 Over-Activation Impairs Reward Motivation on a Progressive-Ratio Schedule of Reinforcement but Has No Effect on Sucrose Preference or Sucrose Consumption

Relevant graphs show mean ± SEM (n = 3 for progressive ratio; n = 4 for sucrose preference). S, sucrose; W, water.

(A) Marmosets were trained to press a circular stimulus on a touchscreen to earn milkshake reward under increasing response demands until breakpoint was reached (2 min with no response).

(B) Task design. The response increment from trial *n* to *n*+1 starts at +1 and doubles every eight trials until a maximum increment of +8 (trials 1–8, responses 1–8; trials 9–16, responses 10–24, etc.).

(C) sgACC/25 over-activation by reducing glutamate reuptake (DHK) decreased the number of responses marmosets made before breakpoint was reached (two-tailed paired t test, p = 0.042).

(D) Response profiles in control and over-activation sessions for each animal. The 2-min timeout period signifying the breakpoint (BrkP) is shaded.

(E) In the sucrose preference test, marmosets were presented with two identical bottles in their home cage: one containing sucrose, and one containing water. A single session lasted 2 hr with measurements taken every 30 min. The first 30-min time point was of *a priori* interest owing to the rapid actions of the intracranial infusions.

(F) Prior to experimental manipulations, marmosets showed a high preference for sucrose during the first 30 min of the session (92.9% ± 1.5%), consuming 32.3 ± 3.2 g sucrose and 2.3 ± 0.2 g water (mean ± SEM).

(G) Cumulative consumption profile in the session prior to experimental manipulations. Marmosets consumed significantly more sucrose at every time point measured (solution [water, sucrose] × time point [four, 30-min time bins], $F_{3,9} = 26.97$, $p < 0.0001$; effect of solution, $p < 0.0001$ at every time point).

(legend continued on next page)

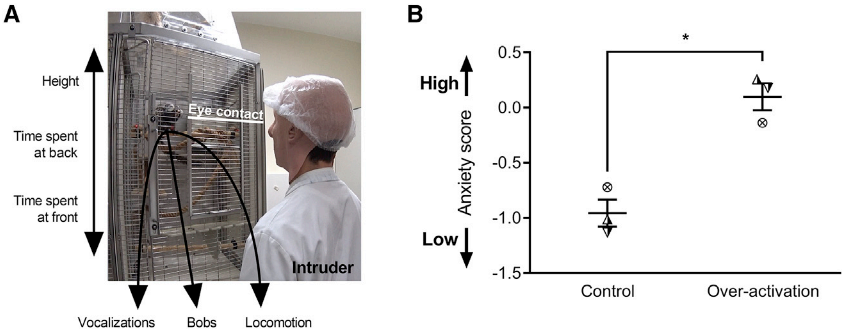

Figure 4. sgACC/25 Over-Activation Does Not Cause a General Blunting in Emotional Arousal

Relevant graphs show mean ± SEM (n = 3).

(A) In the human intruder test, marmosets are divided into a quadrant of their home cage and are confronted with a human intruder who maintains eye contact for 2 min. Marmosets display a range of behaviors in response to the intruder, including vocalizations (tsik, tse, tsik-egg, tse-egg, and egg calls), bobbing (rapid side-to-side movements), and locomotion (translational movements). These behaviors—together with average height, the time spent at the back of the cage, and the time spent at the front of the cage—are measured and loaded onto a single EFA-extracted factor representing anxiety (see Table S5 and STAR Methods).

(B) Anxiety (factor) score following control infusions and over-activation of sgACC/25. Over-activation increased marmosets' anxiety toward the human intruder reflected by an increased anxiety score (two-tailed paired t test, p = 0.047).

arousal is conditioned (anticipatory) or unconditioned (consummatory) cannot be ascertained using the human intruder test. Further experimentation using an aversive Pavlovian conditioning paradigm—equivalent to the appetitive paradigm used above—would address this.

sgACC/25 Over-Activation Is Associated with Metabolic Changes in a Circuit Including dmPFC, dACC, and Insula

To determine the brain regions involved in the blunted anticipatory arousal induced by over-activation of sgACC/25, marmosets (n = 4) underwent ^{18}F-FDG PET imaging to assess regional metabolic activity. Each subject had two counterbalanced scans: one following a saline control infusion, and one following over-activation of sgACC/25 (using DHK) (Figure 5A). In all cases, animals were injected with ^{18}F-FDG and then received a Pavlovian conditioning session in the test apparatus for 30 min before being scanned under anesthesia (Figure 5B). For the voxel-wise analysis, a subtraction image was produced (1) for (over-activation − control) to determine brain regions showing increased activity following sgACC/25 over-activation, and (2) for (control − over-activation) to determine brain regions showing reduced activity. In parallel, we obtained cardiovascular (n = 3 owing to one telemetry probe failure) and behavioral (n = 4) readouts during the PET conditioning session immediately prior to the scan to confirm whether the manipulations replicated the anticipatory impairment described above.

Critically, over-activation of sgACC/25 on the day of scanning replicated the blunting of behavioral and cardiovascular appetitive arousal during CS+ presentation (Figure 5C). The accompanying PET imaging revealed that over-activation of sgACC/25 increased ^{18}F-FDG uptake in sgACC/25 (confirming that the drug manipulation increases metabolism in sgACC/25) together with significant increases in uptake in dorsomedial prefrontal cortex (dmPFC; BA8b, 9), dorsal anterior cingulate cortex (dACC; BA24c), and left ventral insula (Figure 5D). sgACC/25 over-activation also lowered metabolic activity in a brainstem region encompassing components of the serotonergic raphe nuclei (rostral group B9), together with a more caudal region encompassing the nucleus of the solitary tract (NST) and the medullary reticular formation (Figure 5E).

Acute Administration of Ketamine, but Not Citalopram, Reverses Blunted Anticipatory Arousal Induced by Over-Activation of sgACC/25

To determine whether the novel antidepressant ketamine could reverse the blunted anticipatory arousal induced by over-activation of sgACC/25, marmosets (n = 4) received a single intramuscular injection of ketamine (0.5 mg/kg) followed by over-activation of sgACC/25 (using DHK) at 4 hr, 1 day, and 7 days after injection while undergoing behavioral testing on the appetitive Pavlovian conditioning paradigm (Figure 6A). These time points were chosen to coincide with clinical literature showing rapid (4 hr time point) and relatively sustained (1 day and 7 day time points) effects of a single acute administration of ketamine to improve scores on depression scales (Abdallah et al., 2015). In three animals, we also determined the endpoint of ketamine's action.

In a control experiment, ketamine alone (in the absence of sgACC/25 over-activation) had no effect on either autonomic or behavioral components of appetitive arousal compared to vehicle control (Figure S8A). While over-activation of sgACC/25 4 hr following ketamine injection still resulted in blunted anticipatory arousal, over-activation at 1 day and 7 days post-injection did not: despite receiving infusions of DHK, animals showed an MAP rise and head-jerking response selectively to the CS+ (Figure 6C). Therefore, at these time points, ketamine successfully reversed the anticipatory deficit induced by over-activation of sgACC/25. The improvement at 1 day is unlikely to be due to adaptational effects related to a recent DHK infusion (at 4 hr)

(H) Compared to a control infusion of saline, over-activation of sgACC/25 by reducing glutamate reuptake had no effect on sucrose preference in the first 30 min of the session (two-tailed paired t test, p = 0.800).

(I) Over-activation of sgACC/25 had no effect on sucrose or water consumption in the first 30 min of the session (solution × manipulation, $F_{1,3}$ = 1.05, p = 0.381; main effect of manipulation, $F_{1,3}$ = 1.70, p = 0.283).

(J) Across the 2-hr session, over-activation of sgACC/25 had no effect on cumulative sucrose or water consumption (solution × manipulation, F < 1, NS).

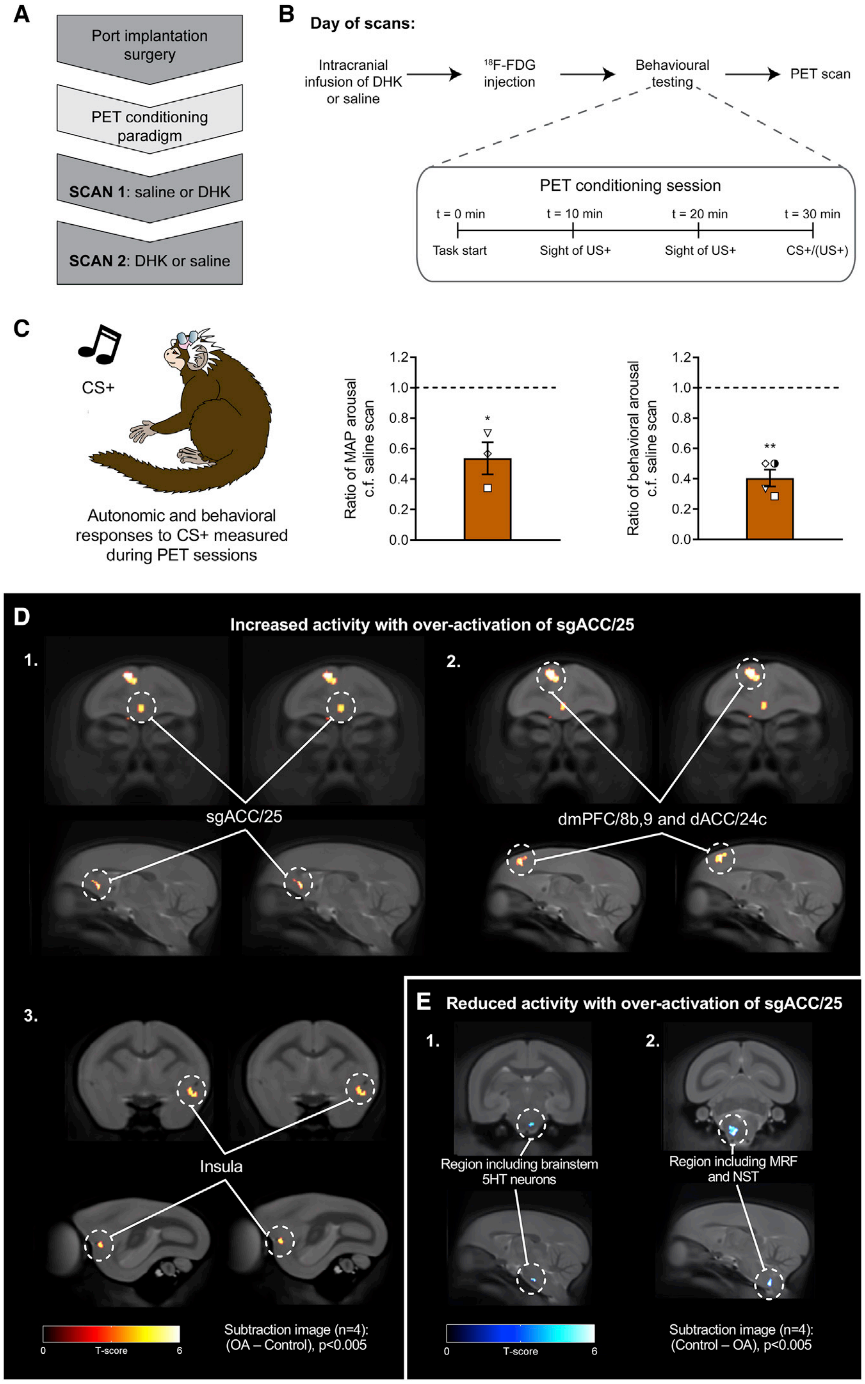

Figure 5. ^{18}F-FDG PET Imaging Revealed Metabolic Changes in a Network of Brain Regions Associated with Reward Processing and Interoception Following sgACC/25 Over-Activation

Relevant graphs show mean ± SEM (n = 3 for cardiovascular arousal; n = 4 for behavioral arousal). n = 4 for all PET images; clusters discussed are significant at the level of $p < 0.005$ with an extent threshold adjusted for search volume of 26 voxels.

(A) Following implantation of a subcutaneous port into the internal jugular vein, marmosets were trained on a modified version of the appetitive Pavlovian conditioning paradigm (see B) in preparation for scanning. Saline control and DHK scans were counterbalanced.

(B) On the day of a scan, animals received an infusion of DHK or saline immediately followed by ^{18}F-FDG injection through the port. The PET conditioning session (inset) lasted 30 min (to increase the sensitivity of ^{18}F-FDG uptake to perturbation by the behavioral paradigm), consisting of two 20 s periods of the sight of reward without access, and a final 20 s CS+ period. During training, the CS+ was followed by a 120 s US+. On scan days, the animals were immediately removed from the apparatus when the CS+ period terminated, anesthetized, and then scanned.

(C) Cardiovascular and behavioral responses were measured during the CS+ period in the PET conditioning sessions immediately prior to scanning. Compared to saline scans, over-activation of sgACC/25 significantly blunted cardiovascular (ratio of MAP response to saline scans; one-sample t test to 1.0, $p = 0.048$) and behavioral (ratio of head-jerk response to saline scans; one sample t test to 1.0, $p < 0.001$) arousal.

(D) Subtraction images calculated from standardized uptake value ratio (SUVR) maps for over-activation (OA) scans—saline control scans, showing brain regions with increased activity following sgACC/25 over-activation. Increased metabolic activity was observed in sgACC/25 (1), together with a region of dorsomedial prefrontal cortex spanning dmPFC/8b,9 and dACC/24c (surviving $p < 0.001$; 2). More caudally, increased metabolic activity was observed in the left ventral insula (3).

(E) Subtraction images calculated from SUVR maps for saline control scans—over-activation scans, showing brain regions with reduced activity following sgACC/25 over-activation. Reduced metabolic activity was observed in a region encompassing brainstem 5HT neurons (1) and, more caudally, brainstem autonomic control centers including the medullary reticular formation (MRF) and the nucleus of the solitary tract (NST; 2).

because we replicate this effect by showing a comparable blunting of cardiovascular and behavioral arousal at the 1-day time point in the subsequent ^{18}F-FDG PET imaging experiment when there was no infusion at 4 hr (see below). In two of the three animals where the end point was assessed, ketamine's action had abrogated by 3 weeks; in the third animal, by 4 weeks (indicated by the return of the over-activation-induced blunting of CS+ arousal).

We also determined the sensitivity of the anticipatory impairment to an acute dose of the first-line SSRI antidepressant citalopram (10 mg/kg; Figure 6D). Acute citalopram has been shown to have rapid and profound effects on marmosets'

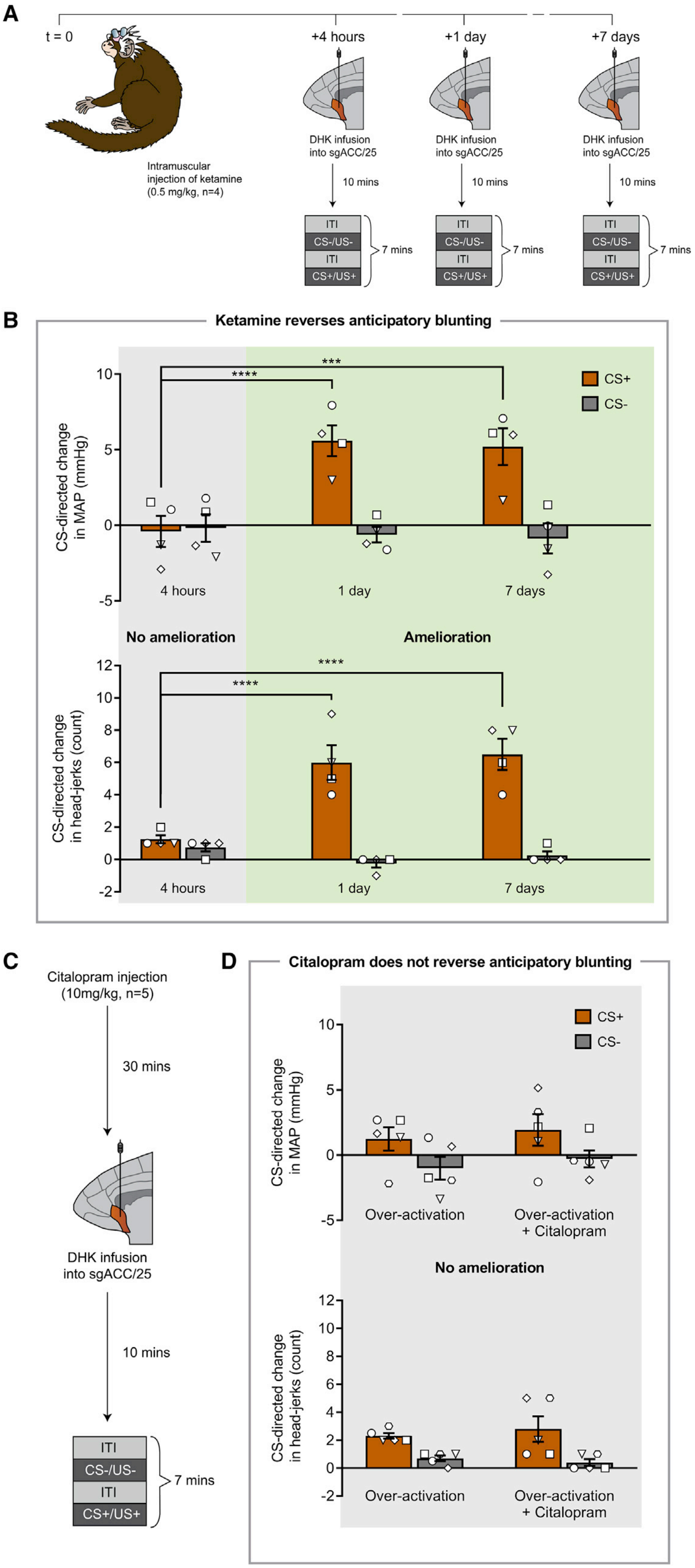

Figure 6. A Single Intramuscular Injection of Ketamine Ameliorates the Cardiovascular and Behavioral Anticipatory Impairment Induced by Over-Activating sgACC/25 in a Time-Dependent Manner, whereas Acute Citalopram Has No Effect

Relevant graphs show mean ± SEM (n = 4 for ketamine study; n = 5 for citalopram study).

(A) Timeline of ketamine study. Marmosets received a single intramuscular injection of ketamine (t = 0) followed by over-activation of sgACC/25 (DHK) 4 hr, 1 day, and 7 days later.

(B) Ketamine had a time-dependent effect to reverse the cardiovascular (time point × CS, $F_{2,12} = 14.71$, $p < 0.001$) and behavioral (time point × CS, $F_{2,12} = 19.59$, $p < 0.001$) aspects of the anticipatory blunting induced by sgACC/25 over-activation (DHK infusions). Compared to control infusions of saline vehicle (not shown), sgACC/25 over-activation 4 hr after ketamine administration still resulted in significant blunting of cardiovascular (manipulation × CS, $F_{1,3} = 60.46$, $p = 0.004$; effect of manipulation on CS+, $p = 0.003$) and behavioral (manipulation × CS, $F_{1,3} = 25.59$, $p = 0.015$; effect of manipulation on CS+, $p = 0.012$) arousal. Over-activation 1 day following ketamine administration evidenced amelioration of the cardiovascular (4 hr versus 1 day: CS+, $p < 0.0001$; CS−, $p = 0.863$) and behavioral (4 hr versus 1 day: CS+, $p < 0.0001$; CS−, $p = 0.371$) impairments compared to 4 hr. Similarly, over-activation 7 days following ketamine administration evidenced amelioration of the cardiovascular (4 hr versus 7 days: CS+, $p < 0.001$; CS−, $p = 0.704$) and behavioral (4 hr versus 7 days: CS+, $p < 0.0001$; CS−, $p = 0.767$) impairments compared to 4 hr.

(C) Timeline of acute citalopram study. Marmosets received a single intramuscular injection of citalopram followed by over-activation of sgACC/25 (DHK) 30 min later.

(D) Compared to sgACC/25 over-activation alone, acute citalopram had no effect on the cardiovascular (manipulation × CS, $F < 1$, NS) or behavioral (manipulation × CS, $F_{1,4} = 1.19$, $p = 0.338$) components of the anticipatory blunting. Compared to control infusions of saline vehicle (not shown), sgACC/25 over-activation with acute citalopram still resulted in significant blunting of cardiovascular (manipulation × CS, $F_{1,4} = 8.74$, $p = 0.042$; effect of manipulation on CS+, $p = 0.016$) and behavioral (manipulation × CS, $F_{1,4} = 462$, $p < 0.0001$; effect of manipulation on CS+, $p < 0.0001$) arousal.

responsivity to a human intruder (Santangelo et al., 2016). In a control experiment, an intramuscular injection of citalopram in the absence of sgACC/25 over-activation had no effect on either autonomic or behavioral components of appetitive arousal (Figure S8B). Unlike ketamine, acute citalopram administration failed to reverse either the autonomic or behavioral components of the over-activation-induced anticipatory deficit, suggesting that it is insensitive to acute SSRI treatment (Figure 6F).

Reversal of Blunted Anticipatory Arousal by Ketamine Is Associated with Reductions in Metabolic Activity within dmPFC, dACC, Insula, and sgACC/25

Marmosets (n = 4) received an additional ^{18}F-FDG PET scan consisting of sgACC/25 over-activation (using DHK) following an injection of ketamine 1 day earlier, coinciding with a time point at which the anticipatory cardiovascular and behavioral blunting was reversed. Subtraction images were computed for (1) (over-activation − [over-activation + ketamine]) to determine brain regions showing decreased metabolic activity following administration of ketamine; and (2) ([over-activation + ketamine] − over-activation) to determine brain regions showing increased activity following administration of ketamine.

Cardiovascular and behavioral data obtained on the day of scanning showed that ketamine administration 1 day prior to over-activation and scanning successfully reversed the DHK-induced anticipatory cardiovascular and behavioral blunting (Figure 7A), thereby replicating our previous findings. This was accompanied by reversal of the metabolic changes within the dmPFC, dACC, and left ventral insula (corresponding to the regions showing elevated activity following sgACC/25 over-activation; Figure 7B). No prefrontal or subcortical regions showed significant increases in activity. To determine whether ketamine normalized metabolic activity to control levels or diminished activity below control levels, a (3) third subtraction image was calculated for (control − [over-activation + ketamine]). Results from this comparison show that while activity in dmPFC/dACC returned to control levels, activity in the left insula was reduced below activity levels observed in the control condition (Figure 7C). Therefore, in the context of sgACC/25 over-activation, ketamine normalizes activity within dmPFC/dACC but deactivates the left insula.

Using the stringent criterion applied in the voxel-based approach, there was no apparent effect of ketamine on metabolic activity in sgACC/25 itself. However, using an atlas-defined region of interest (ROI) (Paxinos et al., 2011), we examined the mean ^{18}F-FDG uptake in sgACC/25 across control, over-activation, and [over-activation + ketamine] conditions to determine if the beneficial effect of ketamine depends—at least in part—on modulation of sgACC/25 activity in response to DHK-induced reductions in glutamate reuptake. Across all four subjects, we found that ketamine administration reduced the increased metabolic activity associated with DHK infusions into sgACC/25 (Table S7). These data suggest that the efficacy of ketamine is related to (likely neuroplastic-mediated) alterations in the responsivity of sgACC/25 to elevated levels of extracellular glutamate.

DISCUSSION

Fractionating Blunted Reward Processing and Its Relevance to Anhedonia

Over-activation of marmoset sgACC/25—achieved using two different methods (reducing glutamate reuptake and increasing pre-synaptic glutamate release)—blunted anticipatory cardiovascular and behavioral responses to a cue predicting food reward (CS+) but did not robustly affect cardiovascular or behavioral responses associated with consumption of the reward (US+). Over-activation by reducing glutamate reuptake through EAAT2 antagonism (DHK) is of particular relevance to depression, since EAAT2 shows reduced expression levels in postmortem cerebral tissue samples of individuals who suffered from depression (Choudary et al., 2005; Miguel-Hidalgo et al., 2010) and in animal models of depression (Zink et al., 2010).

sgACC/25 over-activation also blunted appetitive motivation as assessed by reduced breakpoints on a progressive ratio task. Whether it is possible to separate anticipatory and motivational impairments remains unclear. Impairments in Pavlovian reward anticipation impact instrumental performance through Pavlovian-to-instrumental transfer, conditioned reinforcement, and conditioned approach (Dickinson and Balleine, 1994; Holland, 1977; Mackintosh, 1974). In people with depression, reduced willingness to work for reward is thought to be driven by a primary decrease in anticipatory pleasure (Sherdell et al., 2012). Therefore, motivational impairments may not result from deficits in goal-directed performance per se; rather, from a reduced influence of Pavlovian cues signaling reward that would otherwise support responding.

In contrast, we found no impact of sgACC/25 over-activation on behavioral or cardiovascular consummatory arousal in the Pavlovian conditioning paradigm, nor in the sucrose preference test. This is at odds with the findings of a recent optogenetic study in rodents, which found that activation of mPFC (IL and ventral PL) reduced sucrose preference (Ferenczi et al., 2016). The discrepancy may be related to functional differences between the putative anatomical homologs IL and sgACC/25, to differences in anatomical specificity in the region targeted (in Ferenczi et al., opsin expression was not restricted to IL), or to differences in the type of activation manipulation (here, short-term pharmacological over-activation, whereas in Ferenczi et al., subthreshold depolarization).

The selective anticipatory and motivational impairments illustrate that the transient blunting of reward processing induced by pharmacological over-activation of sgACC/25 possesses face validity when compared to the anhedonia observed in people with depression, who typically show anticipatory and motivational deficits (Klein, 1987; McFarland and Klein, 2009; Treadway and Zald, 2011) rather than consummatory ones (Amsterdam et al., 1987; Arrondo et al., 2015; Berlin et al., 1998; Dichter et al., 2010). The contrasting findings across different reward domains illustrate the importance of careful consideration of the psychological constructs impaired in psychiatric disorders.

The deficits in appetitive arousal were not due to a general blunting of emotional arousal since the same manipulation induced heightened behavioral arousal when marmosets were faced with an uncertain threat represented by a human intruder.

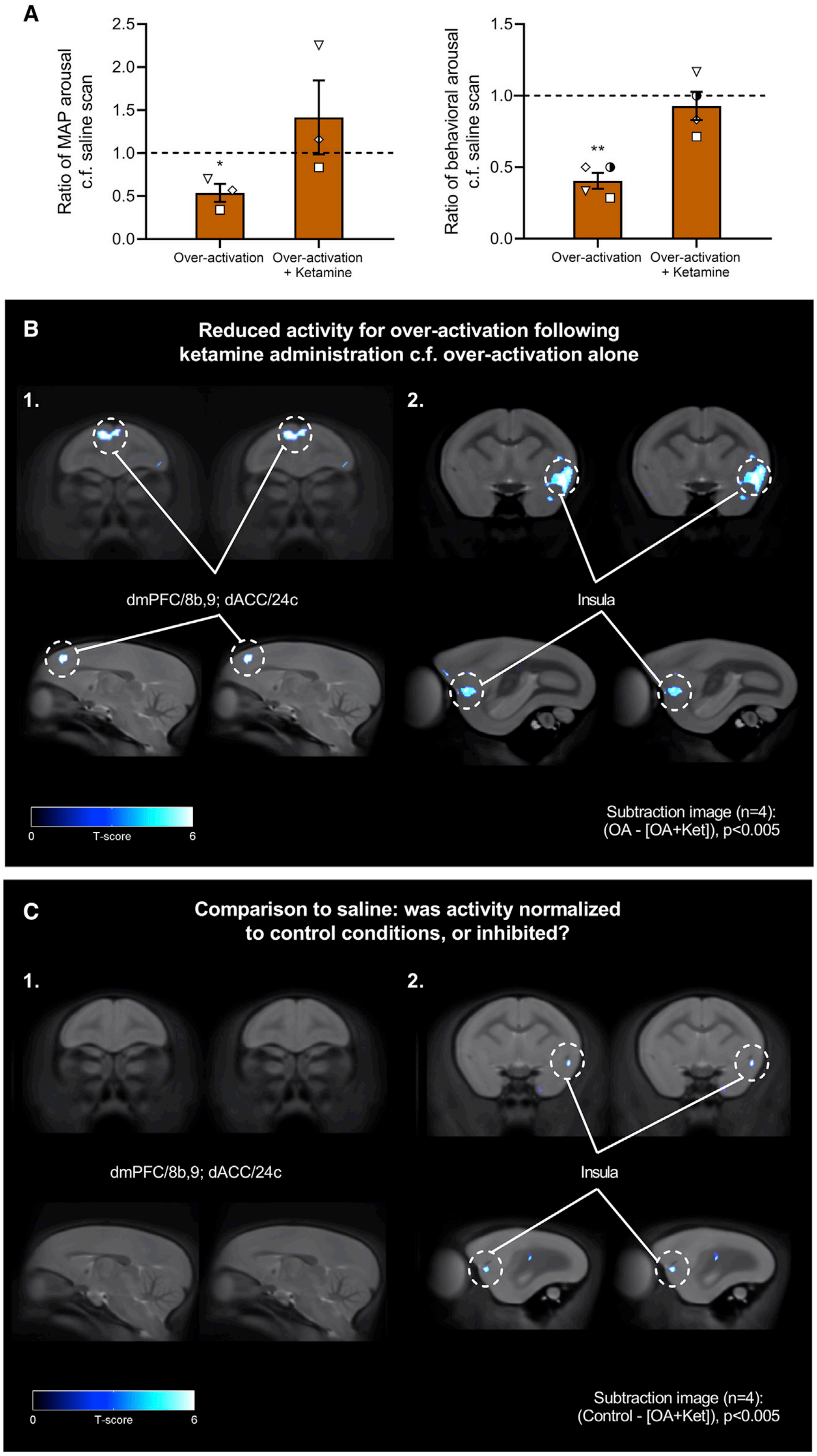

Figure 7. Reversal of Blunted Anticipatory Arousal by Ketamine Is Associated with Metabolic Changes within dmPFC, dACC, Insula, and sgACC/25

Relevant graphs show mean ± SEM (n = 3 for cardiovascular arousal; n = 4 for behavioral arousal). n = 4 for all PET images; clusters discussed are significant at the level of $p < 0.005$ with an extent threshold adjusted for search volume of 26 voxels.

(A) Ketamine administration 1 day earlier ameliorated the blunted cardiovascular arousal associated with over-activation alone, returning cardiovascular arousal to levels no different from the saline scan (one-sample t test on ratio values compared to 1.0, over-activation alone, $p = 0.048$; over-activation + ketamine, $p = 0.435$). Ketamine administration also ameliorated the blunted behavioral arousal associated with over-activation alone, returning behavioral arousal to levels no different from the saline scan (one-sample t test on ratio values compared to 1.0, over-activation alone, $p = 0.007$; over-activation + ketamine, $p = 0.435$).

(B) Subtraction images calculated from SUVR maps for over-activation (OA) scans—[over-activation + ketamine (Ket)] scans, showing brain regions with reduced activation following ketamine administration 1 day earlier. Reversal of blunted anticipatory arousal was associated with reduced activity in dmPFC/8b,9, dACC/24c (1) and left ventral insula (2).

(C) Subtraction images calculated from SUVR maps for control scans—[over-activation + ketamine] scans revealed that activity in the dmPFC/8b,9 and dACC/24c region was no different from control scans, indicating that ketamine had normalized over-activity in these regions to control levels (1). However, activity in the left insula was reduced even compared to control conditions, suggesting that ketamine administration caused deactivation of the insula (2).

This highlights the opposing effects of sgACC/25 over-activity across emotional domains and implicates this region in adapting behavior to emotional context. In addition, these data implicate sgACC/25 over-activity in symptoms of anxiety that commonly manifest in people with depression (Kessler et al., 2003). Indeed, several studies have identified elevated activity in a subgenual region (including area 25) associated with sustained and unpredictable threat (Alvarez et al., 2011; Hasler et al., 2007).

Could an effect of sgACC/25 over-activity on a single construct explain the contrasting effects on appetitive and aversive processing? Hamilton and colleagues have proposed that increased functional connectivity between subgenual regions and the default-mode network seen in people with depression "represents an integration of the self-referential processes supported by the DMN with the affectively laden, behavioral withdrawal processes

associated with sg[ACC]'' linked to rumination and worry (Hamilton et al., 2015). If sgACC/25 over-activity enhances withdrawal, this may manifest as reduced sensitivity to external appetitive cues, together with elevated behavioral withdrawal (increased distance) in the human intruder paradigm. In a previous study, we reported that sgACC/25 *inactivations* elevate parasympathetic tone and reduce anticipatory arousal during a CS predicting a mildly aversive US (Wallis et al., 2017). Elevated parasympathetic tone is associated with affective engagement (rather than withdrawal; Porges, 1992; Thayer and Siegle, 2002), which may facilitate the downregulation of responses to *mildly* aversive stimuli (thereby reducing arousal). Thus, the results of sgACC/25 inactivations in a mildly aversive setting are also consistent with a role for an ''on-line'' sgACC/25 in mediating physiological and behavioral withdrawal.

Circuit-wide Changes Associated with Over-Activation Induced Blunted Reward Processing

PET imaging revealed increased metabolic activity in sgACC/25, dmPFC/dACC, and left ventral insula following over-activation of sgACC/25. Elevated connectivity between similar regions has been observed in depressed populations (Connolly et al., 2013; Hamilton et al., 2015; Sheline et al., 2010), although these changes are seldom related to impairments in reward processing associated with anhedonia. Nevertheless, the increase in dmPFC/dACC activity is consistent with previously reported results of increased activity in a similar region during reward anticipation in currently depressed or remitted patients compared to controls (Dichter et al., 2012; Knutson et al., 2008). Similarly, an over-active insula has been observed during the anticipation of rewards in groups at high risk of depression (Gotlib et al., 2010) and following presentation of positively valenced pictures in people with depression (Mitterschiffthaler et al., 2003).

Reduced activity following sgACC/25 over-activation was seen in a region encompassing brainstem serotonin (5HT) neurons (B9 group of raphé nuclei). The importance of interplay between vmPFC and 5HT neurons has been appreciated in terms of stress controllability (Amat et al., 2005), but more recently a role for 5HT neuronal signaling during reward anticipation has been demonstrated (Li et al., 2016). More caudally, we observed reduced activity in a region including autonomic control centers such as the NST. Several tract tracing studies have identified connectivity between primate vmPFC and autonomic effector regions (Rempel-Clower and Barbas, 1998) through which sgACC/25 can modulate autonomic function (Rudebeck et al., 2014; Wallis et al., 2017). While we did not observe reductions in amygdala and accumbens activity following sgACC/25 over-activation, both the amygdala (Braesicke et al., 2005; Gallagher and Holland, 1994) and accumbens (Datla et al., 2002) have been implicated in various aspects of appetitive Pavlovian conditioning. How these structures may interact with sgACC/25 warrants further investigation.

Ketamine as an Efficacious Treatment for Over-Activation-Induced Blunted Reward Processing

Ketamine reversed the blunted anticipatory cardiovascular and behavioral arousal induced by sgACC/25 over-activation at 1 day and 7 days following administration. This effect was replicated at the 1-day time point in the subsequent PET study, in which ketamine also reduced the DHK-induced increase in metabolic uptake within sgACC/25. The efficacious action of ketamine may therefore be dependent on neuroplastic changes within sgACC/25 that alter the responsivity of the region to elevated levels of extracellular glutamate. This is consistent with an extensive body of literature showing that reductions in activity of a subgenual region are associated with the successful treatment of depressive symptoms (Drevets et al., 2002; Mayberg et al., 2000, 2005).

Downstream of sgACC/25, ketamine also reversed the associated elevations in activity within the dmPFC/dACC and left ventral insula. Activity in the former two regions was normalized, whereas activity in the insula was inhibited below control levels. Normalization of activity in dmPFC/dACC following ketamine differs from a recent clinical study in which the efficacious action of ketamine on symptoms of anhedonia was associated with increased (rather than reduced) metabolism in these regions (Lally et al., 2015). However, the opposing effects may be related to ketamine's actions at different time points: patients in Lally et al. were imaged 2 hr following ketamine administration, whereas in the present study marmosets were imaged 1 day after administration. The rapid versus slow actions of ketamine are associated with different effects on neural circuitry, increasing dACC-mPFC functional connectivity acutely (Grimm et al., 2015), but decreasing it 1 day later (Scheidegger et al., 2012).

Translational Considerations

This study was designed to address several challenges faced in the translation of preclinical studies to humans: specifically, whether cross-species anatomical homology implies analogous function, issues of symptom heterogeneity, and the methodologies used to quantify emotion.

First, concerning cross-species comparisons, the putative anatomical homolog of primate sgACC/25 is rodent IL (Heilbronner et al., 2016; Vogt and Paxinos, 2014), activations of which have variable effects on appetitive behavior (Gasull-Camós et al., 2017; John et al., 2012). However, whether primate/rodent anatomical homology necessarily implies analogous function is far from clear—indeed, we have shown opposite effects on the regulation of negative emotion following inactivations of marmoset sgACC/25 to those seen in rodent IL studies (Wallis et al., 2017). Second, concerning symptom heterogeneity, the impairments in anticipatory and motivational—but not consummatory—domains provide neurobiological evidence for the fractionation of anhedonia into separable subtypes. It is imperative that these subtypes are recognized clinically: people with depression may present with selective impairments in specific aspects of reward processing, with distinct underlying neural mechanisms and hence differing optimal treatment strategies. Finally, anhedonia is a complex emotional construct consisting of behavioral and physiological changes that cannot be adequately measured using single experimental outputs. While informative, studies examining subjective, autonomic, or behavioral components of emotion in isolation fail to account for the complex nature of emotion. Future work must delineate the precise psychological and physiological functions that sgACC/25

subserves in the regulation of positive and negative emotion and isolate the pathophysiological processes that can lead to chronic elevations in sgACC/25 activity associated with mood disorders.

STAR★METHODS

Detailed methods are provided in the online version of this paper and include the following:

- KEY RESOURCES TABLE
- CONTACT FOR REAGENT AND RESOURCE SHARING
- EXPERIMENTAL MODEL AND SUBJECT DETAILS
  - Common marmoset (*Callithrix jacchus*)
- METHOD DETAILS
  - Behavioral testing apparatus and paradigms
  - Surgical procedures
  - Drug treatments
  - PET imaging
  - Post-mortem histological processing
- QUANTIFICATION AND STATISTICAL ANALYSIS
  - Data collection and preliminary analysis
  - Statistical analysis

SUPPLEMENTAL INFORMATION

Supplemental Information includes eight figures and eight tables and can be found with this article online at https://doi.org/10.1016/j.neuron.2018.11.021.

ACKNOWLEDGMENTS

This research was funded by a Wellcome Trust Investigator award (108089/Z/15/Z) to A.C.R. L.A. was funded by a Medical Research Council studentship. H.F.C. was funded by a Medical Research Council Career Development Award (RG62920). We thank Lauren Anderson for histology and behavioral scoring, and Shaun Quah for developing the factor analysis.

AUTHOR CONTRIBUTIONS

Conceptualization, L.A. and A.C.R.; Methodology, L.A., P.L.R.G., H.F.C., and A.C.R.; Formal Analysis, L.A., S.J.S., T.D.F., and Y.T.H.; Investigation, L.A., P.L.R.G., G.J.C., and H.F.C.; Writing – Original Draft, L.A. and A.C.R.; Writing – Review & Editing, L.A. and A.C.R.; Visualization, L.A., S.J.S., T.D.F., and Y.T.H.; Funding Acquisition, A.C.R.; Resources, A.C.R.; Supervision, A.C.R. and H.F.C.

DECLARATION OF INTERESTS

The authors declare no conflicts of interest.

Received: June 25, 2018
Revised: September 10, 2018
Accepted: November 9, 2018
Published: December 4, 2018

REFERENCES

Abdallah, C.G., Sanacora, G., Duman, R.S., and Krystal, J.H. (2015). Ketamine and rapid-acting antidepressants: a window into a new neurobiology for mood disorder therapeutics. Annu. Rev. Med. *66*, 509–523.

Allen, T.A., Narayanan, N.S., Kholodar-Smith, D.B., Zhao, Y., Laubach, M., and Brown, T.H. (2008). Imaging the spread of reversible brain inactivations using fluorescent muscimol. J. Neurosci. Methods *171*, 30–38.

Alvarez, R.P., Chen, G., Bodurka, J., Kaplan, R., and Grillon, C. (2011). Phasic and sustained fear in humans elicits distinct patterns of brain activity. Neuroimage *55*, 389–400.

Amat, J., Baratta, M.V., Paul, E., Bland, S.T., Watkins, L.R., and Maier, S.F. (2005). Medial prefrontal cortex determines how stressor controllability affects behavior and dorsal raphe nucleus. Nat. Neurosci. *8*, 365–371.

Amemori, K., Amemori, S., and Graybiel, A.M. (2015). Motivation and affective judgments differentially recruit neurons in the primate dorsolateral prefrontal and anterior cingulate cortex. J. Neurosci. *35*, 1939–1953.

American Psychiatric Association (2013). Diagnostic and Statistical Manual of Mental Disorders (American Psychiatric Association).

Amsterdam, J.D., Settle, R.G., Doty, R.L., Abelman, E., and Winokur, A. (1987). Taste and smell perception in depression. Biol. Psychiatry *22*, 1481–1485.

Arrondo, G., Murray, G.K., Hill, E., Szalma, B., Yathiraj, K., Denman, C., and Dudas, R.B. (2015). Hedonic and disgust taste perception in borderline personality disorder and depression. Br. J. Psychiatry *207*, 79–80.

Avants, B.B., Epstein, C.L., Grossman, M., and Gee, J.C. (2008). Symmetric diffeomorphic image registration with cross-correlation: evaluating automated labeling of elderly and neurodegenerative brain. Med. Image Anal. *12*, 26–41.

Bechara, A., Tranel, D., Damasio, H., and Damasio, A.R. (1996). Failure to respond autonomically to anticipated future outcomes following damage to prefrontal cortex. Cereb. Cortex *6*, 215–225.

Berlin, I., Givry-Steiner, L., Lecrubier, Y., and Puech, A.J. (1998). Measures of anhedonia and hedonic responses to sucrose in depressive and schizophrenic patients in comparison with healthy subjects. Eur. Psychiatry *13*, 303–309.

Braesicke, K., Parkinson, J.A., Reekie, Y., Man, M.-S., Hopewell, L., Pears, A., Crofts, H., Schnell, C.R., and Roberts, A.C. (2005). Autonomic arousal in an appetitive context in primates: a behavioural and neural analysis. Eur. J. Neurosci. *21*, 1733–1740.

Caldwell, C.A., Watson, C.F.E., and Morris, K.D. (2009). Exploiting flavour preferences of common marmosets to increase palatability of a dry pellet diet. Appl. Anim. Behav. Sci. *116*, 244–249.

Cardinal, R.N., and Aitken, M.R.F. (2010). Whisker: a client-server high-performance multimedia research control system. Behav. Res. Methods *42*, 1059–1071.

Chapman, L.J., Chapman, J.P., and Raulin, M.L. (1976). Scales for physical and social anhedonia. J. Abnorm. Psychol. *85*, 374–382.

Choudary, P.V., Molnar, M., Evans, S.J., Tomita, H., Li, J.Z., Vawter, M.P., Myers, R.M., Bunney, W.E., Jr., Akil, H., Watson, S.J., and Jones, E.G. (2005). Altered cortical glutamatergic and GABAergic signal transmission with glial involvement in depression. Proc. Natl. Acad. Sci. USA *102*, 15653–15658.

Cicchetti, D.V., and Sparrow, S.A. (1981). Developing criteria for establishing interrater reliability of specific items: applications to assessment of adaptive behavior. Am. J. Ment. Defic. *86*, 127–137.

Connolly, C.G., Wu, J., Ho, T.C., Hoeft, F., Wolkowitz, O., Eisendrath, S., Frank, G., Hendren, R., Max, J.E., Paulus, M.P., et al. (2013). Resting-state functional connectivity of subgenual anterior cingulate cortex in depressed adolescents. Biol. Psychiatry *74*, 898–907.

Datla, K.P., Ahier, R.G., Young, A.M.J., Gray, J.A., and Joseph, M.H. (2002). Conditioned appetitive stimulus increases extracellular dopamine in the nucleus accumbens of the rat. Eur. J. Neurosci. *16*, 1987–1993.

Defrise, M., Kinahan, P.E., Townsend, D.W., Michel, C., Sibomana, M., and Newport, D.F. (1997). Exact and approximate rebinning algorithms for 3-D PET data. IEEE Trans. Med. Imaging *16*, 145–158.

Der-Avakian, A., and Markou, A. (2012). The neurobiology of anhedonia and other reward-related deficits. Trends Neurosci. *35*, 68–77.

Dichter, G.S., Smoski, M.J., Kampov-Polevoy, A.B., Gallop, R., and Garbutt, J.C. (2010). Unipolar depression does not moderate responses to the Sweet Taste Test. Depress. Anxiety *27*, 859–863.

Dichter, G.S., Kozink, R.V., McClernon, F.J., and Smoski, M.J. (2012). Remitted major depression is characterized by reward network hyperactivation during

reward anticipation and hypoactivation during reward outcomes. J. Affect. Disord. *136*, 1126–1134.

Dickinson, A., and Balleine, B. (1994). Motivational control of goal-directed action. Anim. Learn. Behav. *22*, 1–18.

Drevets, W.C., Videen, T.O., Price, J.L., Preskorn, S.H., Carmichael, S.T., and Raichle, M.E. (1992). A functional anatomical study of unipolar depression. J. Neurosci. *12*, 3628–3641.

Drevets, W.C., Bogers, W., and Raichle, M.E. (2002). Functional anatomical correlates of antidepressant drug treatment assessed using PET measures of regional glucose metabolism. Eur. Neuropsychopharmacol. *12*, 527–544.

Drevets, W.C., Price, J.L., and Furey, M.L. (2008). Brain structural and functional abnormalities in mood disorders: implications for neurocircuitry models of depression. Brain Struct. Funct. *213*, 93–118.

Dunn, R.T., Kimbrell, T.A., Ketter, T.A., Frye, M.A., Willis, M.W., Luckenbaugh, D.A., and Post, R.M. (2002). Principal components of the Beck Depression Inventory and regional cerebral metabolism in unipolar and bipolar depression. Biol. Psychiatry *51*, 387–399.

Dwyer, D.M. (2012). EPS Prize Lecture. Licking and liking: the assessment of hedonic responses in rodents. Q J Exp Psychol (Hove) *65*, 371–394.

Ebert, D., and Ebmeier, K.P. (1996). The role of the cingulate gyrus in depression: from functional anatomy to neurochemistry. Biol. Psychiatry *39*, 1044–1050.

Fawcett, J., Clark, D.C., Scheftner, W.A., and Gibbons, R.D. (1983). Assessing anhedonia in psychiatric patients. Arch. Gen. Psychiatry *40*, 79–84.

Ferenczi, E.A., Zalocusky, K.A., Liston, C., Grosenick, L., Warden, M.R., Amatya, D., Katovich, K., Mehta, H., Patenaude, B., Ramakrishnan, C., et al. (2016). Prefrontal cortical regulation of brainwide circuit dynamics and reward-related behavior. Science *351*, aac9698.

Ferrari, A.J., Charlson, F.J., Norman, R.E., Patten, S.B., Freedman, G., Murray, C.J.L., Vos, T., and Whiteford, H.A. (2013). Burden of depressive disorders by country, sex, age, and year: findings from the global burden of disease study 2010. PLoS Med. *10*, e1001547.

Fitzgerald, P.B., Laird, A.R., Maller, J., and Daskalakis, Z.J. (2008). A meta-analytic study of changes in brain activation in depression. Hum. Brain Mapp. *29*, 683–695.

Gallagher, M., and Holland, P.C. (1994). The amygdala complex: multiple roles in associative learning and attention. Proc. Natl. Acad. Sci. USA *91*, 11771–11776.

Gasull-Camós, J., Tarrés-Gatius, M., Artigas, F., and Castañé, A. (2017). Glial GLT-1 blockade in infralimbic cortex as a new strategy to evoke rapid antidepressant-like effects in rats. Transl. Psychiatry *7*, e1038.

Gotlib, I.H., Hamilton, J.P., Cooney, R.E., Singh, M.K., Henry, M.L., and Joormann, J. (2010). Neural processing of reward and loss in girls at risk for major depression. Arch. Gen. Psychiatry *67*, 380–387.

Greicius, M.D., Flores, B.H., Menon, V., Glover, G.H., Solvason, H.B., Kenna, H., Reiss, A.L., and Schatzberg, A.F. (2007). Resting-state functional connectivity in major depression: abnormally increased contributions from subgenual cingulate cortex and thalamus. Biol. Psychiatry *62*, 429–437.

Grimm, O., Gass, N., Weber-Fahr, W., Sartorius, A., Schenker, E., Spedding, M., Risterucci, C., Schweiger, J.I., Böhringer, A., Zang, Z., et al. (2015). Acute ketamine challenge increases resting state prefrontal-hippocampal connectivity in both humans and rats. Psychopharmacology (Berl.) *232*, 4231–4241.

Hamilton, J.P., Farmer, M., Fogelman, P., and Gotlib, I.H. (2015). Depressive rumination, the default-mode network, and the dark matter of clinical neuroscience. Biol. Psychiatry *78*, 224–230.

Harvey, P.-O., Pruessner, J., Czechowska, Y., and Lepage, M. (2007). Individual differences in trait anhedonia: a structural and functional magnetic resonance imaging study in non-clinical subjects. Mol. Psychiatry *12*, 703–, 767–775.

Hasler, G., Fromm, S., Alvarez, R.P., Luckenbaugh, D.A., Drevets, W.C., and Grillon, C. (2007). Cerebral blood flow in immediate and sustained anxiety. J. Neurosci. *27*, 6313–6319.

Heilbronner, S.R., Rodriguez-Romaguera, J., Quirk, G.J., Groenewegen, H.J., and Haber, S.N. (2016). Circuit-based corticostriatal homologies between rat and primate. Biol. Psychiatry *80*, 509–521.

Holland, P.C. (1977). Conditioned stimulus as a determinant of the form of the Pavlovian conditioned response. J. Exp. Psychol. Anim. Behav. Process. *3*, 77–104.

Ito, H., Kawashima, R., Awata, S., Ono, S., Sato, K., Goto, R., Koyama, M., Sato, M., and Fukuda, H. (1996). Hypoperfusion in the limbic system and prefrontal cortex in depression: SPECT with anatomic standardization technique. J. Nucl. Med. *37*, 410–414.

John, C.S., Smith, K.L., Van't Veer, A., Gompf, H.S., Carlezon, W.A., Jr., Cohen, B.M., Öngür, D., and Bechtholt-Gompf, A.J. (2012). Blockade of astrocytic glutamate uptake in the prefrontal cortex induces anhedonia. Neuropsychopharmacology *37*, 2467–2475.

Keedwell, P.A., Andrew, C., Williams, S.C.R., Brammer, M.J., and Phillips, M.L. (2005). The neural correlates of anhedonia in major depressive disorder. Biol. Psychiatry *58*, 843–853.

Keedwell, P., Drapier, D., Surguladze, S., Giampietro, V., Brammer, M., and Phillips, M. (2009). Neural markers of symptomatic improvement during antidepressant therapy in severe depression: subgenual cingulate and visual cortical responses to sad, but not happy, facial stimuli are correlated with changes in symptom score. J. Psychopharmacol. (Oxford) *23*, 775–788.

Keller, M.B., Klein, D.N., Hirschfeld, R.M., Kocsis, J.H., McCullough, J.P., Miller, I., First, M.B., Holzer, C.P., 3rd, Keitner, G.I., Marin, D.B., et al. (1995). Results of the DSM-IV mood disorders field trial. Am. J. Psychiatry *152*, 843–849.

Kessler, R.C., Berglund, P., Demler, O., Jin, R., Koretz, D., Merikangas, K.R., Rush, A.J., Walters, E.E., and Wang, P.S.; National Comorbidity Survey Replication (2003). The epidemiology of major depressive disorder: results from the National Comorbidity Survey Replication (NCS-R). JAMA *289*, 3095–3105.

Klein, D. (1987). Anhedonia and Affect Deficit States (PMA Publishing).

Knutson, B., Bhanji, J.P., Cooney, R.E., Atlas, L.Y., and Gotlib, I.H. (2008). Neural responses to monetary incentives in major depression. Biol. Psychiatry *63*, 686–692.

Lally, N., Nugent, A.C., Luckenbaugh, D.A., Ameli, R., Roiser, J.P., and Zarate, C.A. (2014). Anti-anhedonic effect of ketamine and its neural correlates in treatment-resistant bipolar depression. Transl. Psychiatry *4*, e469.

Lally, N., Nugent, A.C., Luckenbaugh, D.A., Niciu, M.J., Roiser, J.P., and Zarate, C.A., Jr. (2015). Neural correlates of change in major depressive disorder anhedonia following open-label ketamine. J. Psychopharmacol. (Oxford) *29*, 596–607.

Li, Y., Zhong, W., Wang, D., Feng, Q., Liu, Z., Zhou, J., Jia, C., Hu, F., Zeng, J., Guo, Q., et al. (2016). Serotonin neurons in the dorsal raphe nucleus encode reward signals. Nat. Commun. *7*, 10503.

Mackintosh, N.J. (1974). The Psychology of Animal Learning (Academic Press).

Mayberg, H.S., Lewis, P.J., Regenold, W., and Wagner, H.N., Jr. (1994). Paralimbic hypoperfusion in unipolar depression. J. Nucl. Med. *35*, 929–934.

Mayberg, H.S., Brannan, S.K., Tekell, J.L., Silva, J.A., Mahurin, R.K., McGinnis, S., and Jerabek, P.A. (2000). Regional metabolic effects of fluoxetine in major depression: serial changes and relationship to clinical response. Biol. Psychiatry *48*, 830–843.

Mayberg, H.S., Lozano, A.M., Voon, V., McNeely, H.E., Seminowicz, D., Hamani, C., Schwalb, J.M., and Kennedy, S.H. (2005). Deep brain stimulation for treatment-resistant depression. Neuron *45*, 651–660.

McCabe, C., Woffindale, C., Harmer, C.J., and Cowen, P.J. (2012). Neural processing of reward and punishment in young people at increased familial risk of depression. Biol. Psychiatry *72*, 588–594.

McFarland, B.R., and Klein, D.N. (2009). Emotional reactivity in depression: diminished responsiveness to anticipated reward but not to anticipated punishment or to nonreward or avoidance. Depress. Anxiety *26*, 117–122.

Miguel-Hidalgo, J.J., Waltzer, R., Whittom, A.A., Austin, M.C., Rajkowska, G., and Stockmeier, C.A. (2010). Glial and glutamatergic markers in depression, alcoholism, and their comorbidity. J. Affect. Disord. *127*, 230–240.

Mitterschiffthaler, M.T., Kumari, V., Malhi, G.S., Brown, R.G., Giampietro, V.P., Brammer, M.J., Suckling, J., Poon, L., Simmons, A., Andrew, C., and Sharma, T. (2003). Neural response to pleasant stimuli in anhedonia: an fMRI study. Neuroreport *14*, 177–182.

Myers-Schulz, B., and Koenigs, M. (2012). Functional anatomy of ventromedial prefrontal cortex: implications for mood and anxiety disorders. Mol. Psychiatry *17*, 132–141.

Parsaik, A.K., Singh, B., Khosh-Chashm, D., and Mascarenhas, S.S. (2015). Efficacy of ketamine in bipolar depression: systematic review and meta-analysis. J. Psychiatr. Pract. *21*, 427–435.

Paxinos, G., Watson, C., Petrides, M., Rosa, M., and Tokuno, H. (2011). The Marmoset Brain in Stereotaxic Coordinates (Academic Press).

Porges, S.W. (1992). Autonomic regulation and attention. In Attention and Information Processing in Infants and Adults: Perspectives from Human and Animal Research (Lawrence Erlbaum Associates, Inc), pp. 201–223.

Pryce, C.R., Dettling, A.C., Spengler, M., Schnell, C.R., and Feldon, J. (2004). Deprivation of parenting disrupts development of homeostatic and reward systems in marmoset monkey offspring. Biol. Psychiatry *56*, 72–79.

Reekie, Y.L., Braesicke, K., Man, M.S., and Roberts, A.C. (2008). Uncoupling of behavioral and autonomic responses after lesions of the primate orbitofrontal cortex. Proc. Natl. Acad. Sci. USA *105*, 9787–9792.

Rempel-Clower, N.L., and Barbas, H. (1998). Topographic organization of connections between the hypothalamus and prefrontal cortex in the rhesus monkey. J. Comp. Neurol. *398*, 393–419.

Roberts, A.C., Robbins, T.W., and Everitt, B.J. (1988). The effects of intradimensional and extradimensional shifts on visual discrimination learning in humans and non-human primates. Q. J. Exp. Psychol. B *40*, 321–341.

Roberts, A.C., Tomic, D.L., Parkinson, C.H., Roeling, T.A., Cutter, D.J., Robbins, T.W., and Everitt, B.J. (2007). Forebrain connectivity of the prefrontal cortex in the marmoset monkey (Callithrix jacchus): an anterograde and retrograde tract-tracing study. J. Comp. Neurol. *502*, 86–112.

Rudebeck, P.H., Putnam, P.T., Daniels, T.E., Yang, T., Mitz, A.R., Rhodes, S.E.V., and Murray, E.A. (2014). A role for primate subgenual cingulate cortex in sustaining autonomic arousal. Proc. Natl. Acad. Sci. USA *111*, 5391–5396.

Santangelo, A.M., Ito, M., Shiba, Y., Clarke, H.F., Schut, E.H., Cockcroft, G., Ferguson-Smith, A.C., and Roberts, A.C. (2016). Novel primate model of serotonin transporter genetic polymorphisms associated with gene expression, anxiety and sensitivity to antidepressants. Neuropsychopharmacology *41*, 2366–2376.

Scheidegger, M., Walter, M., Lehmann, M., Metzger, C., Grimm, S., Boeker, H., Boesiger, P., Henning, A., and Seifritz, E. (2012). Ketamine decreases resting state functional network connectivity in healthy subjects: implications for antidepressant drug action. PLoS ONE *7*, e44799.

Sheline, Y.I., Price, J.L., Yan, Z., and Mintun, M.A. (2010). Resting-state functional MRI in depression unmasks increased connectivity between networks via the dorsal nexus. Proc. Natl. Acad. Sci. USA *107*, 11020–11025.

Sherdell, L., Waugh, C.E., and Gotlib, I.H. (2012). Anticipatory pleasure predicts motivation for reward in major depression. J. Abnorm. Psychol. *121*, 51–60.

Spijker, J., Bijl, R.V., de Graaf, R., and Nolen, W.A. (2001). Determinants of poor 1-year outcome of DSM-III-R major depression in the general population: results of the Netherlands Mental Health Survey and Incidence Study (NEMESIS). Acta Psychiatr. Scand. *103*, 122–130.

Thayer, J.F., and Siegle, G.J. (2002). Neurovisceral integration in cardiac and emotional regulation. IEEE Eng. Med. Biol. Mag. *21*, 24–29.

Treadway, M.T., and Zald, D.H. (2011). Reconsidering anhedonia in depression: lessons from translational neuroscience. Neurosci. Biobehav. Rev. *35*, 537–555.

Tye, K.M., Mirzabekov, J.J., Warden, M.R., Ferenczi, E.A., Tsai, H.-C., Finkelstein, J., Kim, S.-Y., Adhikari, A., Thompson, K.R., Andalman, A.S., et al. (2013). Dopamine neurons modulate neural encoding and expression of depression-related behaviour. Nature *493*, 537–541.

Uher, R., Perlis, R.H., Henigsberg, N., Zobel, A., Rietschel, M., Mors, O., Hauser, J., Dernovsek, M.Z., Souery, D., Bajs, M., et al. (2012). Depression symptom dimensions as predictors of antidepressant treatment outcome: replicable evidence for interest-activity symptoms. Psychol. Med. *42*, 967–980.

Vogt, B.A., and Paxinos, G. (2014). Cytoarchitecture of mouse and rat cingulate cortex with human homologies. Brain Struct. Funct. *219*, 185–192.

Wallis, C.U., Cardinal, R.N., Alexander, L., Roberts, A.C., and Clarke, H.F. (2017). Opposing roles of primate areas 25 and 32 and their putative rodent homologs in the regulation of negative emotion. Proc. Natl. Acad. Sci. USA *114*, E4075–E4084.

Zink, M., Vollmayr, B., Gebicke-Haerter, P.J., and Henn, F.A. (2010). Reduced expression of glutamate transporters vGluT1, EAAT2 and EAAT4 in learned helpless rats, an animal model of depression. Neuropharmacology *58*, 465–473.

STAR★METHODS

KEY RESOURCES TABLE

REAGENT or RESOURCE	SOURCE	IDENTIFIER
Antibodies		
Rabbit polyclonal to c-fos primary antibody	Abcam	Cat #ab190289
Goat anti-Rabbit IgG H&L [Biotin] secondary antibody	Abcam	Cat #ab6720, RRID: AB_954902
Chemicals, Peptides, and Recombinant Proteins		
Baclofen	Sigma Aldrich	Cat #BP028, CAS 1134-47-0
Citalopram HBr	Sigma Aldrich	Cat #C7861, CAS 59729-32-7
Muscimol	Sigma Aldrich	Cat #M1523, CAS 2763-96-4
Naloxone HCl	Sigma Aldrich	Cat #N7758, CAS 51481-60-8
CGP52432	Tocris	Cat #1246/10, CAS 139667-74-6
Dihydrokainic acid	Tocris	Cat #0111, CAS 52497-36-6
LY341495	Tocris	Cat #1209, CAS 201943-63-7
Ketamine HCl [Ketavet]	Henry Schein	Cat #PFKET03, CAS 1867-66-9
Experimental Models: Organisms/Strains		
Common marmoset (*Callithrix jacchus*)	University of Cambridge Marmoset Breeding Colony	N/A
Software and Algorithms		
MATLAB	MathWorks	R2013a, RRID: SCR_001622
Power Director	CyberLink Corporation	11.0.0.2027
Prism	GraphPad	8.00.178, RRID: SCR_002798
Spike2	Cambridge Electronic Design	8.11a, RRID: SCR_000903
Whisker	Cambridge University Technical Services Ltd.	4.6

CONTACT FOR REAGENT AND RESOURCE SHARING

Requests for resources, reagents or questions about methods should be directed to Lead Contact, Professor Angela C. Roberts (acr4@cam.ac.uk).

EXPERIMENTAL MODEL AND SUBJECT DETAILS

Common marmoset (*Callithrix jacchus*)

Eleven marmosets (*Callithrix jacchus*, eight females and three males), bred on-site at the University of Cambridge Marmoset Breeding Colony, were housed in male/female pairs (males were vasectomized). Seven were experimentally naive. Four marmosets had varying amounts of aversive Pavlovian discrimination training prior to the study. For a summary of subjects used in the experiments described in the manuscript, see Tables 1 and S1.

Animals were kept in a 12-hour light-dark cycle (lights on at 7am, lights off at 7pm) in a controlled environment of 22 ± 1°C temperature and 50 ± 1% humidity. Their cages (280 × 120 × 98cm) contained a nest-box together with a variety of environmental enrichment aids including suspended ladders, wooden branches, ropes and boxes. Animals had *ad libitum* access to water. On weekdays, subjects were fed 20 g of MP.E1 primate diet (Special Diet Services, Essex, UK) and two pieces of carrot. On week-ends, their diet was supplemented with fruit, rusk, malt loaf, eggs, bread and treats. All procedures were carried out in accordance with the UK Animals (Scientific Procedures) Act 1986 as amended in 2012, under project licences 80/2225 and 70/7618. In addition, the University of Cambridge Animal Welfare and Ethical Review Body (AWERB) provided ethical approval of the project licence and its amendments, as well as individual studies and procedures via delegation of authorization to the NACWO for individual study plans.

METHOD DETAILS

Behavioral testing apparatus and paradigms

Carry-box

Animals were trained to enter a transparent Perspex carry-box (240 × 230 × 200mm) in which they were transported to the behavioral testing apparatus. The Perspex carry-box was placed inside the test chamber, and the marmoset remained inside this box during testing. The carry-box had two circular windows (diameter 30mm) on opposite sides.

Discriminative conditioning

Behavioral testing took place within a sound-attenuated testing chamber in a dark room. The chamber was lit by an 3W bulb (house-light), located in the middle of the ceiling. Two electrically controlled food-box units were attached to the left and right walls of the internal frame of the apparatus. A telemetry receiver, used to record cardiovascular data, was concealed beneath the floor of the apparatus. Each food-box was cylindrical (internal diameter 52mm and length 51mm). When the carry-box was fitted into the internal frame of the apparatus, the positions of the carry-box windows were aligned with the food-boxes. The inside of each food-box could be illuminated by a 28V, 0.04W encased light bulb. Access to both food-boxes was restricted by black and opaque Perspex doors, which could be opened remotely to allow access. The chamber contained computer-controlled speakers through which auditory stimuli could be played, and three cameras used to record the animal during testing using video software (CyberLink, Power Director, CyberLink Corp.). The video display was shown on a monitor outside of the testing apparatus meaning the animal could be observed by the experimenter during testing. The apparatus was controlled by the Whisker control system (Cardinal and Aitken, 2010) and in-house software.

Prior to conditioning, all marmosets were habituated to the sight and sound of the food-box doors opening and closing. During these sessions, high incentive food (several pieces of marshmallow) was presented in either the left or right food-box and the door of the food-box was opened after 120 s. When the animal stopped showing a startle response (i.e., rearing and jumping) to the opening of the door and started consuming marshmallow within 40 s of its opening, they were advanced to conditioning sessions. The mean number of habituation sessions was 10 ± 1 (mean ± SEM).

Marmosets were then exposed to two novel auditory cues and the cardiovascular arousal response (MAP) was measured. The cue that produced the smallest arousal response became the CS+ and the cue that produced the largest arousal response became the CS-. The animals were then trained on an appetitive Pavlovian conditioning paradigm: the CS+ was associated with food reward (US+; marshmallow, net weight approximately 5.8-6.2g) and the CS- was associated with no reward (US-). Marshmallows were chosen as the food reward since marmosets invariably favor them over other types of food (Caldwell et al., 2009). A trial consisted of a 20 s CS period during which one of the cues was played. At the end of this period, one of the food-boxes would open, accompanied by the house-light offset, the onset of the food-box light and presentation of either an empty box (US-) or the high-incentive food reward (US+). The auditory CS continued to be played for the entire 120 s duration of the US period. In multiple-trial sessions, the offset of the US period was indicated by termination of the CS, closure of the black opaque food-box door and onset of the house-light. If the trial was the last in a session, all lights were turned off at the end of the US period indicating session termination. The intervals between trials were pseudorandomly varied between 70-110 s. There were either one or two trials in each session with no more than one CS+/US+ trial; if present, the CS+/US+ trial was always the final trial. Thus, a session could consist of a single CS-/US- or CS+/US+ trial, two CS-/US- trials or one CS-/US- trial and one CS+/US+ trial. Infusions were always conducted on sessions containing both CS-/US- and CS+/US+ trial types which lasted 460 s in total. Behavioral and cardiovascular measurements were analyzed both during the CS and US periods as well as the 20 s baseline (BL) periods immediately prior to the onset of the CS.

Progressive ratio

Behavioral testing took place within a sound-attenuated chamber in a dark room, identical to the chamber described in Discriminative conditioning but without food-box units. When the carry-box was fitted into the internal frame of the apparatus and the door removed, the marmosets had access to a touchscreen (Campden Instruments, Loughborough, UK). The stimulus presented on the screen was a white circle (diameter of 75mm) displayed to the left or right of the central spout via the Whisker control system. When appropriate, a reward of cooled banana milkshake (Nestlé) was delivered through a centrally placed spout for 5 s. A brief tone (0.5 s, 80dB) played from speakers at the back of the chamber signaled reward availability.

During training, marmosets were first familiarized with the delivery of banana milkshake from a spout in the testing apparatus. They were then trained to respond to stimuli presented on a touchscreen for reward. Once marmosets were reliably and accurately making $\geq$ 30 responses in 10 min to a green square presented to the left or right of the licker (see Roberts et al., 1988), the stimulus was changed to a white circle presented at a fixed location (the animal's preferred side). Fixed ratio (FR) response schedules were then introduced to familiarize marmosets with the requirement to make repeated responses for reward. Marmosets progressed from FR1 → FR2 → FR3 → FR5 → FR7 response schedules when their performance at each level was stable. After FR7, marmosets were trained on a progressive ratio schedule of reinforcement taken from (Pryce et al., 2004). In this schedule, the response increment from trial n to n+1 starts at +1 and doubles every eight trials until a maximum increment of +8 (trials 1-8: responses 1-8; trials 9-16: responses 10-24 etc.). After two minutes of inactivity (or a session length of 30 min), marmosets 'timed-out' and were removed from the apparatus. The total number of responses marmosets made prior to timing-out was considered the breakpoint.

Sucrose preference test

The sucrose preference test was carried out in animals' home cages.

During a testing session, animals were divided into the top left or top right quadrant of the cage and the nest-box was removed. Marmosets were presented with two bottles identical in appearance: one water bottle and one containing sucrose (25 g in 250 g water). Each session lasted two hours, and from session to session the left-right position of the two bottles was varied. Every 30 min, an experimenter briefly removed each bottle and weighed it, before replacing it in the same position. The amount of sucrose consumed and sucrose preference (sucrose/[sucrose+water]) was measured over the session. Once marmosets achieved stable sucrose preference > 90% over two sessions, experimental manipulations took place.

Human intruder test

Human intruder tests were carried out in animals' home cages.

During testing sessions, animals were divided in the top right quadrant of the cage and the nest-box was removed. After 8 min of habituation to separation, an unfamiliar intruder entered the room. The intruder wore different latex masks to disguise their face. The intruder stood 40cm from the front of the cage and stared at the marmoset for 2 min. Behavior was recorded using a video camera and microphone. Several measures were scored: time spent at the front (TSAF) of the cage, time spent at the back (TSAB) of the cage, average height, bobbing (rapid side-to-side body movements), vocalizations (egg calls, tsik call, tsik-egg calls, and tse-egg calls) and locomotion.

PET conditioning

Marmosets undergoing ^{18}F-FDG PET scanning were trained on a modified version of the Pavlovian conditioning paradigm. The length of the session was increased from 460 s to 1800s to increase the sensitivity of ^{18}F-FDG uptake to perturbation by the behavioral paradigm. Marmosets were habituated to the increased length of the session by gradually increasing the time spent in the testing apparatus over approximately 12 habituation sessions (12 ± 1.4, mean ± SEM). During a standard PET conditioning session, the opaque door of the rewarded food-box opened at 600 s and 1200s for 20 s revealing the high-incentive food reward, after which it closed again. During this period, marmosets could see the reward without being able to access it – the sight of reward is also known to act as an appetitive CS (Braesicke et al., 2005). At 1660s, the CS+ auditory cue was played for 20 s after which the rewarded food-box opened for 120 s as before (US+). The CS+ continued to be played for the entire 120 s of the US+ period. During the habituation and PET conditioning sessions, marmosets underwent the entire PET procedure. This included being taken to the rooms used for intracranial infusions, intramuscular and ^{18}F-FDG injections. Marmosets were also given mock injections before testing to minimize the effects of handling on the day of the scan. All animals received this on every testing session from at least 4 days before the first scan until the final scan.

Marmosets received at least 5 of these training sessions before undergoing the first PET scan. Owing to the requirement for low blood glucose concentration and anesthesia during PET scanning, marmosets were unable to consume food reward on the day of the scan. Therefore, in sessions conducted on the day of scanning, animals were removed from the apparatus at 1680s (immediately after experiencing the CS+) and the session was terminated without a US+.

Surgical procedures

Eleven animals underwent two aseptic surgical procedures: one to implant intracerebral cannulae targeting either sgACC/25 alone or both sgACC/25 and pgACC/32, and one to implant a telemetric blood pressure (BP) monitor into the descending aorta. Four of these animals underwent a third procedure to implant a vascular access port for administration of ^{18}F-FDG. Animals had 7-10 days of recovery following all surgical procedures.

Cannulation surgery

Marmosets were pre-medicated with ketamine hydrochloride (Ketavet; 0.10ml of a 100mg solution, i.m.; Henry Schein, Melville, NY) before being given a long lasting non-steroidal anti-inflammatory analgesic (Carprieve; 0.03ml of 50mg/mL carprofen, s.c.; Pfizer, Kent, UK). They were intubated and maintained on 2.0%–2.5% isoflurane in 0.3L/min O_2 and placed into a stereotaxic frame modified for the marmoset (David Kopf, Tujunga, CA). HR, SpO_2, breathing rate and CO_2 saturation were all monitored by pulse-oximetry and capnography (Microcap Handheld Capnograph, Oridion Capnography, MA, USA) and core body temperature was monitored by a rectal thermometer (TES-1319 K-type digital thermometer, TES Electrical Electronic Corp, Taipei, Taiwan). Cannulae (Plastics One, Roanoke, VA) were implanted into sgACC/25 (26-gauge double cannulae, 7.0mm long, 1.0 to 1.4mm apart, anteroposterior [AP] +14, lateromedial [LM] ± 0.5) and pgACC/32 (26-gauge double cannulae, 2.0 to 3.5mm long, 1.0 to 1.2mm apart, AP +17, LM ± 0.5 at 30° AP angle) adjusted *in situ* according to cortical depth (Roberts et al., 2007). Postoperatively, animals received the analgesic meloxicam (0.1ml of a 1.5mg/mL oral suspension; Boehringer Ingelheim) for 3 days. Cannulae were cleaned every week (and caps and cannula blockers changed) to ensure the cannulae remained patent and the site free from infection.

Telemetry probe surgery

Animals were anaesthetized as before. The descending aorta was visualized within the abdominal cavity and the probe catheter of a telemetric BP transmitter (Data Sciences International [DSI], St. Paul, MN, USA) was implanted into the aorta just above the aortic bifurcation as described previously (Braesicke et al., 2005). All monkeys received meloxicam as before in addition to prophylactic treatment with amoxicillin and clavulanic acid (Synulox; 50mg/mL solution, Pfizer, Kent, UK) one day before and for 6 days after telemetry surgery.

Port implant surgery

A vascular access port (Solomon Scientific, Skokie, IL, USA) was implanted into the animal to allow swift subcutaneous injection of ^{18}F-FDG. Animals were anaesthetized as before and placed on to the surgical table in a prone position. An incision was made below the shoulder-blades perpendicular to the axis of the spine where the port would be placed, and a second incision was made on the neck to expose the jugular vein. A catheter attached to the port was threaded under the skin from the back toward the neck. The port was placed in a skin pocket created by the first incision. A small incision was made in the jugular vein to insert the open end of the catheter in the direction of the heart. The catheter was glued to the vein with Vetbond (M3 Animal Care Products, MN, USA) and the incisions on the back and neck were sutured. All animals received meloxicam and amoxicillin/clavulanic acid perioperatively as before.

Drug treatments

For all sterile drug treatments, the marmoset was held gently in a researcher's hand.

Central infusions

For central infusions, the caps and cannula blockers were removed from the guide, and the site was cleaned with 70% alcohol. A sterile injector (Plastics One) connected to a 2μl gas tight syringe in a syringe pump was inserted into the guide cannula. Bilateral infusions were carried out over 2 min at a rate dependent on the drug being infused. Following the infusion, the injector was left in place for a further minute to allow the drug to diffuse before injector removal. Sterile cannula blockers and caps were replaced, and the marmoset was returned to its cage for a pre-treatment time dependent on the drug being infused.

Intramuscular injections

For intramuscular injections, the injection site (located on the lateral aspect of the thigh) was cleaned with alcohol and injected with a drug or an equal volume of saline vehicle before testing.

See Table S8 for a summary of doses and timings for centrally and peripherally administered compounds in this study. Of these, DHK is the drug used most extensively in this study. Its pre-treatment time of 10 min was chosen based on work in rodents showing maximal effects within 15 min of infusion, which return to control levels within 30-45 min (John et al., 2012).

PET imaging

Each animal selected to undergo PET scanning received three ^{18}F-FDG PET scans with a microPET Focus-220 scanner (Concorde Microsystems, Knoxville, TN) with the first scan approximately 2 weeks after port implant surgery and an interval between scans of approximately 2 weeks. On the day of a scan, animals did not receive breakfast to lower blood glucose concentration and hence increase the transport of ^{18}F-FDG into brain tissue, thereby increasing the cerebral ^{18}F-FDG signal-to-noise ratio. The animal received an infusion of either saline vehicle or DHK approximately 10 min prior to a bolus injection of approximately 70MBq of ^{18}F-FDG administered subcutaneously via the vascular access port. They were then taken to the test apparatus and after 30 min of the behavioral paradigm described previously, the animal was anaesthetized as described above. The animal was then placed on a heat-pad on the scanner bed and attached to monitoring equipment. HR, SpO_2 and breathing rate were monitored continuously. The bed of the scanner was then positioned to locate the brain in the center of the PET scanner field-of-view, where both sensitivity and resolution are optimal. For consistency, PET data acquisition started 70 min after the ^{18}F-FDG injection and lasted for 45 min. The energy and coincidence timing windows used were 350-650keV and 6 ns, respectively.

The list mode PET data were histogrammed into 9 × 5 min 4D sinograms and then reconstructed using Fourier rebinning (FORE, Defrise et al., 1997) followed by the 2D ordered subsets expectation maximization (OSEM) algorithm installed on the scanner (6 iterations, 16 subsets). As post-injection transmission scanning was not feasible, attenuation correction used a mean non-attenuation corrected ^{18}F-FDG image to determine a body outline, within which a uniform attenuation coefficient (0.096cm^{-1}) was ascribed. This was combined with a standard attenuation map of the carbon fiber bed determined from transmission scanning. The combined attenuation map was forward projected using software installed on the scanner to produce an attenuation correction factor sinogram, and image reconstruction was repeated with attenuation correction applied. Corrections were also applied for random coincidences, dead-time, normalization, scatter, sensitivity and decay.

Post-mortem histological processing

Assessment of cannula placement

Animals were pre-medicated with ketamine hydrochloride before being euthanized with pentobarbital sodium (Dolethal; 1ml of a 200mg/mL solution, i.e.; Merial Animal Health, Essex, UK). Animals were then perfused transcardially with 300ml 0.1M PBS, followed by 300ml of 4% paraformaldehyde fixative solution over approximately 15 min. The brain was removed and left in the 4% paraformaldehyde fixative solution overnight before being transferred to 0.01M PBS-azide solution for at least 48 hours. Finally, the brains were transferred to 30% sucrose solution for at least 48 hours for cryoprotection. Brains were then sectioned on a freezing microtome (coronal sections; 40-60μm), mounted on slides and stained with cresyl-violet. The sections were viewed under a Leitz DMRD microscope (Leica Microsystems, Wetzlar, Germany). The cannula locations for each animal were schematized onto drawings of standard marmoset brain coronal sections together with estimated infusion spread (Figure S1A) which was estimated at 0.5-1.0mm (based on Allen et al., 2008).

c-fos *expression*

One hour prior to perfusion, an animal was infused with DHK into left sgACC/25 and saline vehicle contralaterally. The animal was euthanized, perfused and brains sectioned as above before the tissue was immunohistochemically processed for *c-fos* expression. Sections were washed for 3 × 10 min in 0.01M PBS and incubated for 10 min in 10% methanol/10% H_2O_2 v/v solution to inhibit endogenous peroxidase activity. Sections were then washed and blocked for two hours with 3% normal goat serum before being incubated overnight with the primary antibody (1:2000 Rabbit polyclonal to c-fos; ab190289, Abcam, Cambridge, UK). The following day, sections were washed and incubated for two hours with the secondary antibody (1:500 Goat Anti-Rabbit IgG H&L [Biotin]; ab6720, Abcam). After secondary incubation, sections were incubated in an avidin/biotin complex solution for 30 min (RTU ABC reagent, Vector Labs, Peterborough, UK) and then reacted in 3,3′ diaminobenzidine (DAB) chromagen for 15 s (ImmPact DAB SK-4105, Vector Labs). Following DAB reaction, sections were transferred to ice-cold PBS and mounted on gelatin-coated slides. Slides were

dehydrated, coverslipped using DPX mounting medium (Sigma-Aldrich, MI, US) and visualized using a Leitz DMRD microscope. See Figure S1B for *c-fos* expression.

QUANTIFICATION AND STATISTICAL ANALYSIS

Data collection and preliminary analysis

Discriminative conditioning: telemetry data collection and preliminary analysis

BP data were continuously transmitted by the implanted probe to a receiver for offline analysis using Spike2 (Version 8.11a, CED) as described previously (Braesicke et al., 2005). Any outliers and recording failures in the data were removed (BP values above 200mmHg or below 0mmHg, or other abnormal spikes). Data collection was reliable overall, but data gaps of less than 0.4 s were replaced by cubic spline interpolation and gaps of more than 0.4 s were treated as missing values. Systolic (s)BP events were extracted as local maxima from each cardiac cycle, and diastolic (d)BP events extracted as local minima. The HR was calculated using the time interval between adjacent sBP events. MAP was calculated from adjacent systolic and diastolic values using the formula MAP = dBP + 1/3(sBP-dBP). A mean MAP and HR value was calculated over the 20 s anticipatory CS period. The 20 s immediately preceding each CS period served as its baseline for comparison purposes: the CS-directed autonomic measures were calculated as the difference between the mean value for CS period and the mean value for the baseline period. The principal measure for the consummatory period was the US-directed MAP response, calculated as the difference in MAP response between the US period (calculated as a mean MAP response after the animals began consuming the reward) and 20 s CS period.

Discriminative conditioning: behavioral data collection and preliminary analysis

Behavior during the discrimination was recorded and subsequently scored by an experimenter and a blinded research assistant (correlation between scorers: R^2 = 0.59, considered ‘excellent’ (Cicchetti and Sparrow, 1981). Behaviors were scored separately during the anticipatory period and consummatory period. The anticipatory CS period behaviors scored were CS-directed orienting behaviors known as ‘head-jerks’ (Reekie et al., 2008). The number of anticipatory head-jerks was compared to the value in the 20 s preceding baseline period to give a CS-directed score. Baseline and CS period locomotion was also scored to determine if sgACC/25 over-activation had any effect on locomotion levels, measured as the total time (s) an animal spent in motion (movement of all four limbs plus movement about the body axis; see Table S3). The consummatory behavior scored was the amount of reward consumed across the 120 s period (grams).

Pre-processing of PET data

MR imaging of the animals was not possible due to the cannulae implanted in the brain, preventing the use of MR-based spatial normalization. Instead, first, the mean FDG image of each scan was manually, rigidly registered to an FDG brain template produced from another FDG study in the colony that included MR imaging. The FDG brain template was constructed by averaging n = 21 FDG images transformed to template space using registration transformations obtained by warping MR images (co-registered to the FDG images) to an MR template. Second, for each subject, the three FDG scans rigidly registered to the FDG template were averaged, the resultant image was non-rigidly registered (affine and non-linear) to the FDG template using ANTS (Avants et al., 2008), and this transformation was applied to each of the three rigidly registered FDG scans. Use of a single spatial normalization transformation per subject rather than per FDG scan was adopted after it was found – using the n = 21 FDG scans with MR – that this approach provided ROI PET values with a higher correlation to those obtained using MR-based spatial normalization (R^2 = 0.89 versus R^2 = 0.87).

For each scan, an SUVR map was created for voxel-wise analysis by dividing the mean PET image by a cerebellum ROI value. Normalization by the cerebellum signal was designed to minimize the confounding influence of inter-scan differences in tracer availability, plasma glucose concentration, the effect of anesthesia on cerebral blood flow and metabolism, and basal cerebral glucose metabolism.

An sgACC/25 ROI was manually defined in template space on the MR template according to the Paxinos et al. marmoset atlas (Paxinos et al., 2011), averaged across hemispheres, manually edited and mirrored about the mid-line to provide symmetric left and right ROIs. The mean SUVR value in each ROI was determined by overlaying the ROIs onto the SUVR map.

Statistical analysis

Where appropriate, data were inputted into GraphPad Prism v8.00.178 for Windows (GraphPad Software, La Jolla, CA) for statistical analysis. Significance was set at $\alpha = 0.05$ in all cases. Raw data are available from the authors.

Discriminative conditioning: illustrating discrimination

To illustrate successful discrimination between CS+ and CS-, a two-tailed paired t test was conducted on CS-directed cardiovascular and behavioral measurements in sessions prior to drug manipulations. Cardiovascular discrimination between US+ and US- was assessed in the same way.

Discriminative conditioning: drug manipulations

CS measurements taken during drug manipulation sessions were compared to infusions of saline vehicle using a two-way repeated-measures ANOVA of the form $C_2 \times M_2$ where *C* is a within-subject factor with two levels (CS type: CS+, CS-) and *M* is a within-subject factor with two levels (manipulation type: saline, drug). Significant interactions were subjected to Sidak’s multiple comparisons test applied to vehicle versus drug data for CS+ and CS- (to ascertain whether there were changes in responses to the

CS+ selectively, CS- selectively or both). Absolute MAP values during the baseline and CS+ periods were also compared using a two-way repeated-measures ANOVA of the form $P_2 \times M_2$ where *P* is a within-subject factor with two levels (phase: baseline, CS+) and *M* is a within-subject factor with two levels (manipulation type: saline, drug). US+ measurements taken during drug manipulations were compared to infusions of saline vehicle using a two-tailed paired t test.

To determine if DHK and CGP/LY manipulations changed absolute locomotion levels, baseline locomotion and CS locomotion were compared across saline and drug conditions using two-way repeated-measures ANOVAs. To determine if changes in locomotion were correlated with changes in MAP, CS-directed locomotion changes were correlated with CS-directed MAP changes across saline, DHK and CGP/LY infusion sessions into sgACC/25. R^2 values were calculated to ascertain the strength of correlation between MAP change and locomotion change across infusion types.

Discriminative conditioning: ketamine/citalopram study

For the ketamine study, cardiovascular and behavioral measurements were subjected to a two-way repeated-measures ANOVA of the form $C_2 \times T_3$ where *C* is a within-subject factor with two levels (CS type) and *T* represents time point with three levels (4 hours, 1 day, 7 days). Significant interactions were subjected to Sidak's multiple comparisons test, applied to vehicle versus drug data across CS type. Ketamine control data were analyzed using a two-way repeated-measures ANOVA of the form $C_2 \times M_2$ as described above. Cardiovascular and behavioral data from citalopram control and citalopram manipulation studies were analyzed using two-way repeated-measures ANOVAs of the form $C_2 \times M_2$ as described above.

Progressive ratio

For control and drug sessions, a percentage change in the number of responses at breakpoint was calculated compared to the previous day. A two-tailed paired t test was conducted on percentage change values for control versus drug sessions.

Sucrose preference test: first 30-min

A two-tailed paired t test was conducted to compare preference values between control and drug conditions over the first 30-min window. Sucrose and water consumption over the first 30-min window were analyzed using a two-way repeated-measures ANOVA of the form $M_2 \times S_2$ where *M* is a within-subject factor with two levels (manipulation type) and *S* is a within-subject factor with two levels (solution type).

Sucrose preference test: two-hour session

To compare the effect of drug manipulations on cumulative consumption of sucrose versus cumulative consumption of water across the entire two-hour testing session, data were subjected to a three-way repeated-measures ANOVA of the form $M_2 \times S_2 \times T_4$ (*M*: within-subject factor of two levels [manipulation type]; *S*: within-subject factor of two levels [solution type]; *T*: within-subject factor of four levels [time window]). In the case of naloxone manipulations, planned comparisons were made between naloxone and control manipulations at each time point for water and sucrose solutions separately using Fisher's LSD test.

Human intruder test

An exploratory factor analysis (EFA) was carried out on human intruder test scores obtained as part of a screening procedure on 171 marmosets from the University of Cambridge Marmoset Breeding Colony, to predict the extent to which the different behaviors in the test are driven by an underlying latent variable. The EFA included: TSAF, TSAB, average height, proportion of time spent in locomotion, number of bobs, egg calls, tsik call, tsik-egg calls, and tse-egg calls. In total, nine factors were identified. Based on the point of inflection on a scree plot, a single factor was extracted accounting for 39.7% of the total variance in behavior. The loading of individual behaviors onto this factor is shown in Table S5; the pattern of loading suggests this factor represents the marmosets' anxiety toward the human intruder. For example, a higher factor (anxiety) score is associated with increased distance from the human intruder and lower locomotion, together with increased vigilance in the form of head bobbing and tse-egg vocalizations.

PET scanning

SPM8 (Wellcome Trust Institute for Neurology, UCL, UK) was used for voxel-wise analysis. A general linear model was configured with covariates for subject and condition (saline control versus over-activation versus [over-activation + ketamine]) and changes in activity were tested with Student's t test at each voxel. Prior to estimating the model, images were smoothed with a filter size of $1mm^3$ using a locally adapted Gaussian kernel to include only those voxels inside a brain mask. In mitigation against type I errors expected due to multiple comparisons, an adjusted p value of $p < 0.005$ was applied with an extent threshold adjusted for search volume of 26 voxels.

For analysis of SUVR values in the sgACC/25 ROI across control, over-activation and [over-activation + ketamine] conditions, data were subjected to a two-way repeated-measures ANOVA of the form $M_3 \times H_2$ where *M* is a within-subject factor with two levels (manipulation type) and *H* is a within-subject factor with two levels (hemisphere). Planned comparisons were made between control versus over-activation and over-activation versus [over-activation + ketamine] values using Fisher's LSD test.

PET conditioning

A ratio was calculated comparing CS-directed cardiovascular and behavioral measures during the CS+ for control scans versus over-activation scans and control scans versus [over-activation + ketamine] scans. A one sample t test was performed to determine whether the ratio significantly different from a hypothetical value of 1.0 (no difference).

Neuron, Volume *101*

Supplemental Information

Fractionating Blunted Reward Processing

Characteristic of Anhedonia by Over-Activating

Primate Subgenual Anterior Cingulate Cortex

Laith Alexander, Philip L.R. Gaskin, Stephen J. Sawiak, Tim D. Fryer, Young T. Hong, Gemma J. Cockcroft, Hannah F. Clarke, and Angela C. Roberts

Supplemental

Figures

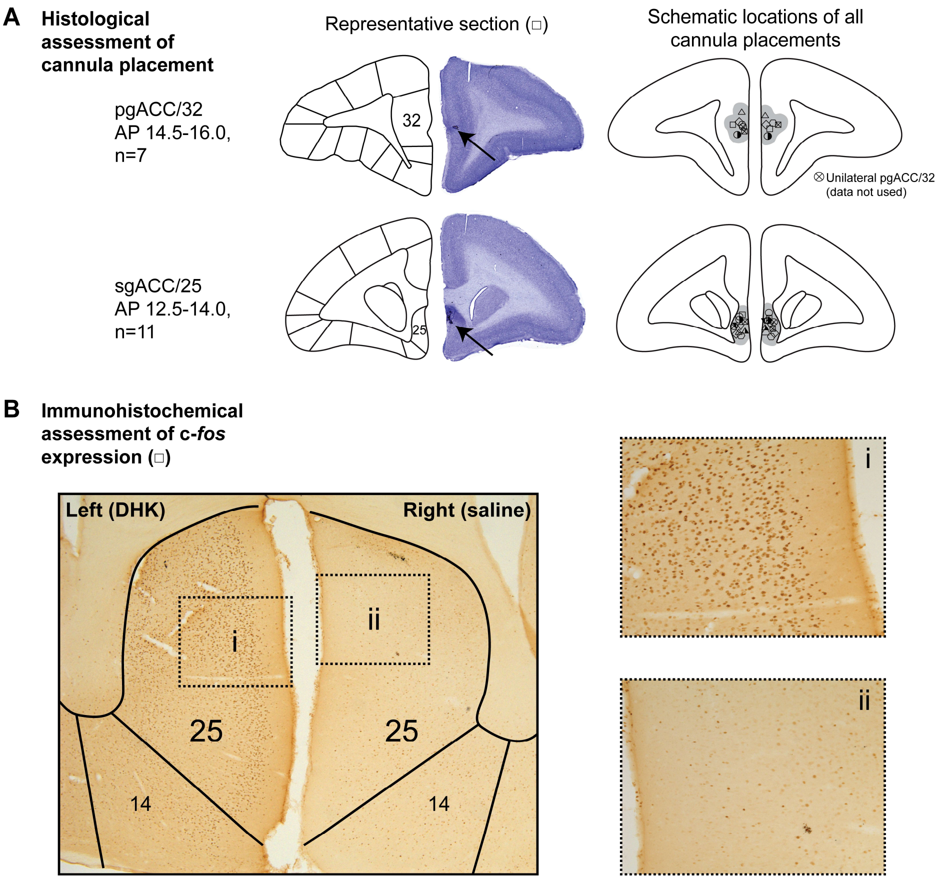

Figure S1. Verification of cannula placements and immunohistochemical assessment of c-*fos* expression. Related to Table 1 and all figures. A Histological assessment of cannula placement using cresyl violet staining. Representative sections are shown with pgACC/32 and sgACC/25 cannulation sites indicated by an arrow. No damage was noted in any animals apart from the small area of gliosis used to pinpoint cannula placement. A schematic diagram shows the cannula placements for all monkeys reported in the manuscript together with estimated infusion spread in grey (approximately 0.5-1.0mm). **B** c-*fos* expression was assessed at the end of the study in one marmoset (□) following DHK infusion in left sgACC/25 and saline infusion in right sgACC/25. DHK infusions (inset i) – but not saline infusions (inset ii) – caused robust c-*fos* expression in sgACC/25. This subject had the highest number of infusions out of all subjects reported in this manuscript, showing that the cortex remains sensitive to drug manipulations.

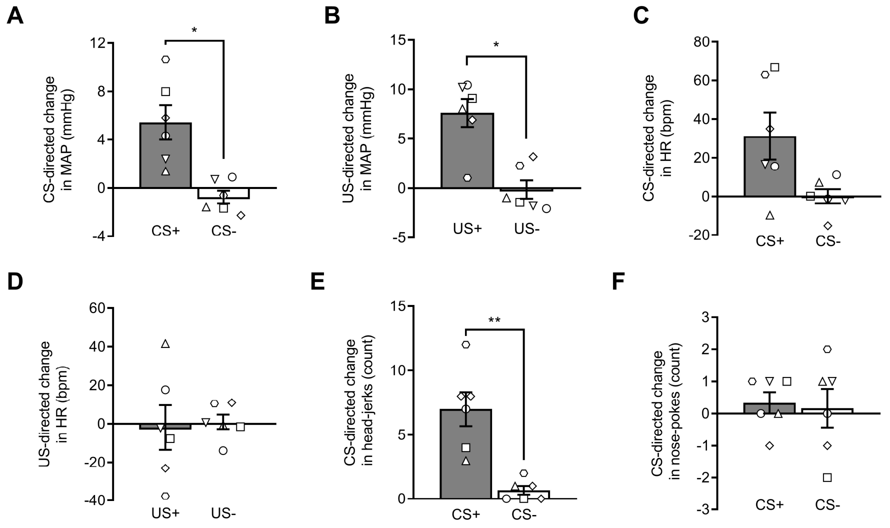

Figure S2. **Cardiovascular and behavioral measures associated with appetitive Pavlovian conditioning. Related to Figure 1.** Relevant graphs show mean ± SEM (n=6). **A** Animals showed CS-directed (CS minus baseline) anticipatory MAP responses to the CS+ but not the CS- (two-tailed paired *t*-test, p=0.013). **B** Animals showed US-directed (US minus CS) consummatory MAP responses to the US+ but not the US- (two-tailed paired *t*-test, p=0.017). **C** Heart rate (HR) discrimination was evident in five of six animals, although the response magnitudes were variable resulting in a non-significant discrimination (two-tailed paired *t*-test, p=0.077). **D** HR responses during the consummatory period were highly variable, with no discrimination evident between US+ and US- (two-tailed paired *t*-test, p=0.848). **E** Behaviorally, animals showed rapid orienting responses (head-jerks) to the CS+ but not the CS- (two tailed paired *t*-test, p=0.003). **F** Nose-pokes were measured as a US-directed conditioned behavior during the CS period. Nose-poking behavior was highly variable and did not discriminate between CS type (two-tailed paired *t*-test, p=0.793).

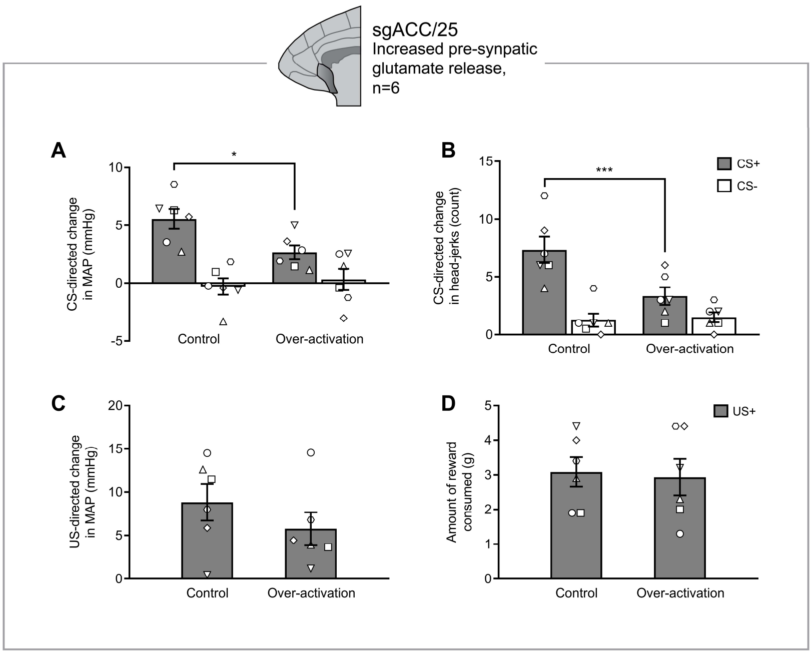

Figure S3. Effects of over-activation of sgACC/25 using CGP/LY. Related to Figure 2. Relevant graphs show mean ± SEM (n=6). **A** sgACC/25 over-activation by increasing pre-synaptic glutamate release (CGP/LY) blunted anticipatory cardiovascular arousal in a CS-dependent manner (manipulation × CS, $F_{1,5}$=14.39, p=0.013) decreasing responding to the CS+ but not the CS- (effect of manipulation: CS+, p=0.014; CS-, p=0.634). **B** The same manipulation blunted anticipatory behavioral arousal in a CS-dependent manner (manipulation × CS, $F_{1,5}$=48.08, p=0.001) decreasing responding to the CS+ but not the CS- (effect of manipulation: CS+, p<0.001; CS-, p=0.839). **C** There was no significant effect on consummatory cardiovascular arousal during the US+ (two-tailed paired *t*-test, p=0.129). **D** There was no significant effect on reward consumption during the US+ (two-tailed paired *t*-test, p=0.665).

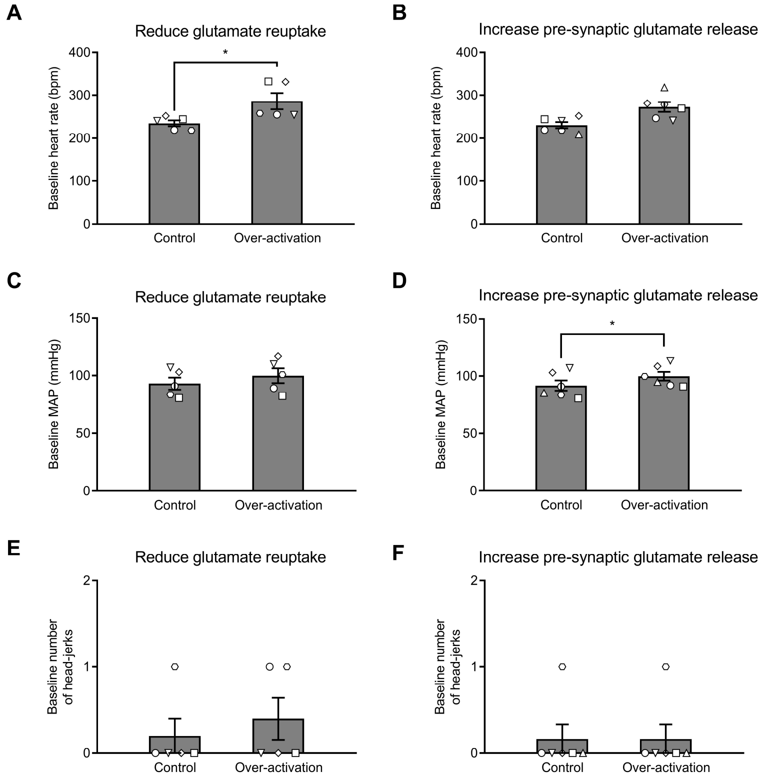

Figure S4. Baseline (20s pre-CS) period effects of sgACC/25 over-activation on HR and MAP. Related to Figures 2 and S3. Relevant graphs show mean ± SEM (n=5 for reduced glutamate reuptake; n=6 for increased pre-synaptic glutamate release). **A** Over-activation of sgACC/25 by reducing glutamate reuptake (DHK) increased baseline HR (two-tailed paired *t*-test, p=0.029). **B** Over-activation of sgACC/25 by increasing pre-synaptic glutamate release (CGP/LY) tended to increase baseline HR (two-tailed paired *t*-test, p=0.051). **C** Reducing glutamate reuptake had no significant effect on baseline MAP (two-tailed paired *t*-test, p=0.097). **D** Increasing pre-synaptic glutamate release increased baseline MAP (two-tailed paired *t*-test, p=0.014). **E** Reducing glutamate reuptake had no significant effect on baseline head-jerk numbers (two-tailed paired *t*-test, p=0.374). **F** Increasing pre-synaptic glutamate release had no effect on baseline head-jerk numbers (values identical across conditions).

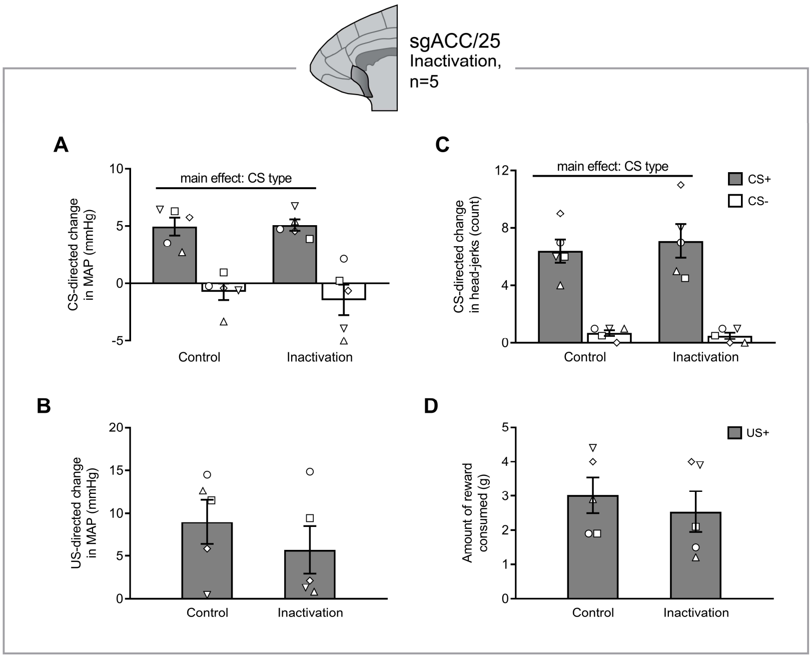

Figure S5. sgACC/25 inactivation had no effect on appetitive anticipatory or consummatory arousal. Related to Figure 2. Relevant graphs show mean ± SEM (n=5). **A** sgACC/25 inactivation using $GABA_A/GABA_B$ receptor agonists (muscimol/baclofen, MB) had no effect on anticipatory cardiovascular arousal (manipulation × CS, $F<1$, NS; main effect of CS, $F_{1,4}=31.76$, p=0.005). **B** The same manipulation had no effect on anticipatory behavioral arousal (manipulation × CS, $F_{1,4}=1.59$, p=0.276; main effect of CS, $F_{1,4}=35.27$, p=0.004). **C** There was no significant effect on consummatory cardiovascular arousal during the US+ (two-tailed paired *t*-test, p=0.226). **D** There was no significant effect on reward consumption during the US+ (two-tailed paired *t*-test, p=0.220).

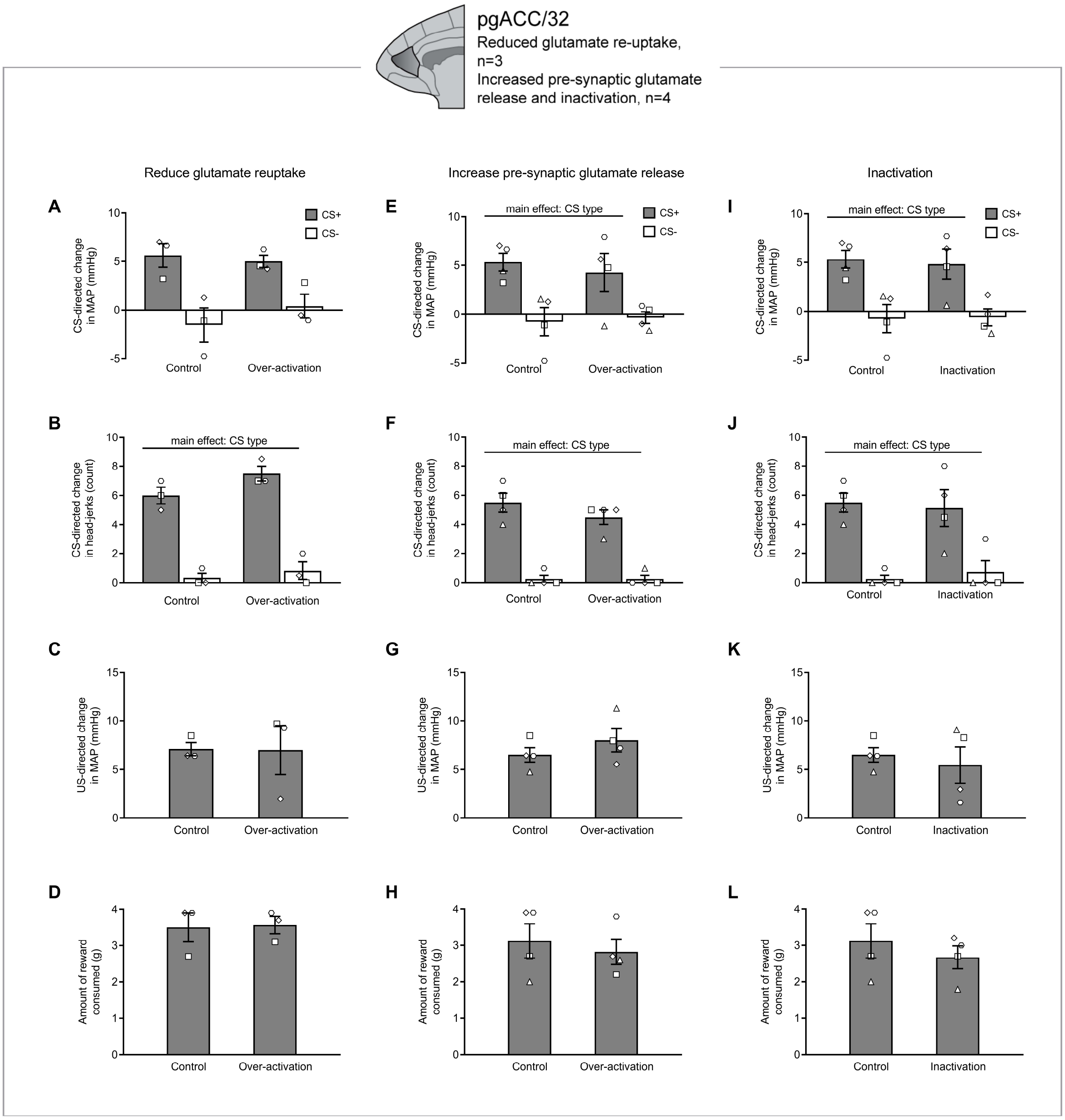

Figure S6. Neither pgACC/32 over-activation nor pgACC/32 inactivation impairs anticipatory or consummatory arousal. Related to Figure 2. Relevant graphs show mean ± SEM (n=4 for inactivation and increased pre-synaptic release; n=3 for reduced glutamate re-uptake). **A** PgACC/32 over-activation by reducing glutamate reuptake (DHK) had no effect on anticipatory cardiovascular arousal (manipulation × CS, $F_{1,2}$=7.77, p=0.108; main effect of CS, $F_{1,2}$=10.07, p=0.087). **B** Reducing glutamate reuptake had no effect on anticipatory behavioral arousal (manipulation × CS, F<1, NS; main effect of CS, $F_{1,2}$=342.3, p=0.003). **C** Reducing glutamate reuptake had no effect on consummatory cardiovascular arousal during the US+ (two-tailed paired t-test, p=0.966). **D** Reducing glutamate reuptake had no effect on reward consumption during the US+ (two-tailed paired t-test, p=0.742). **E** PgACC/32 over-activation by increasing pre-synaptic glutamate release (CGP/LY) had no effect on anticipatory cardiovascular arousal (manipulation × CS, $F_{1,3}$=1.55, p=0.301; main effect of CS, $F_{1,3}$=11.45, p=0.043). **F** Increasing pre-synaptic glutamate release had no effect on anticipatory behavioral arousal (manipulation × CS, $F_{1,3}$=6.00, p=0.092; main effect of CS, $F_{1,3}$=63.71, p=0.004). **G** Increasing pre-synaptic glutamate release had no effect on consummatory cardiovascular arousal

during the US+ (two-tailed paired *t*-test, p=0.450). **H** Increasing pre-synaptic glutamate release had no effect on reward consumption during the US+ (two-tailed paired *t*-test, p=0.484). **I** PgACC/32 inactivation using $GABA_A/GABA_B$ receptor agonists (MB) had no effect on anticipatory cardiovascular arousal (manipulation × CS, $F<1$, NS; main effect of CS, $F_{1,3}=16.50$, p=0.027). **J** Inactivation had no effect on anticipatory behavioral arousal (manipulation × CS, $F_{1,3}=1.77$, p=0.275; main effect of CS, $F_{1,3}=62.85$, p=0.004). **K** Inactivation had no effect on consummatory cardiovascular arousal during the US+ (two-tailed paired *t*-test, p=0.646). **L** Inactivation had no significant effect on reward consumption during the US+ (two-tailed paired *t*-test, p=0.122).

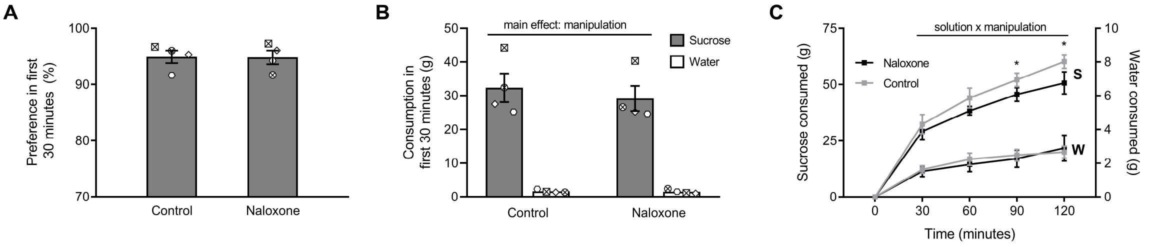

Figure S7. Effects of naloxone, an opioid receptor antagonist, on performance in the sucrose preference test. Related to Figure 3. Relevant graphs show mean ± SEM (n=4). S, sucrose; W, water. **A** Compared to a control injection of saline, the opioid antagonist naloxone had no effect on sucrose preference in the first 30 minutes of the session (two-tailed paired *t*-test, p=0.952). **B** Naloxone reduced both water and sucrose consumption in the first 30 minutes of the session (solution × manipulation, $F_{1,3}$=4.25, p=0.131; main effect of manipulation, $F_{1,3}$=18.00, p=0.024). **C** Across the two-hour session, naloxone reduced cumulative sucrose consumption but not cumulative water consumption (solution × manipulation, $F_{0.532,1.597}$=30.47, p=0.046). Planned comparisons conducted on sucrose and water measurements at each timepoint using Fisher's LSD test revealed a significant decrease in sucrose consumption following naloxone treatment at 90 minutes (p=0.010) and 120 minutes (p=0.024), with no significant effect on water consumption at any timepoint.

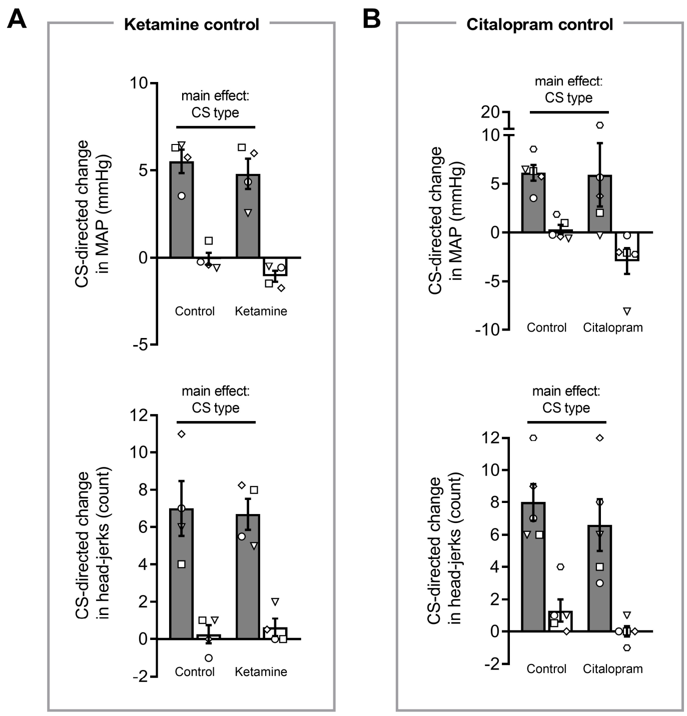

Figure S8. Ketamine and citalopram control experiments (with no intracerebral infusions). Related to Figure 6. Relevant graphs show mean ± SEM (n=4 for ketamine control, n=5 for citalopram control). **A** Ketamine alone (CS-/CS+ sessions in between DHK timepoints) had no effect on cardiovascular (manipulation × CS, $F<1$, NS; main effect of CS maintained, $F_{1,3}=86.50$, p=0.003) or behavioral (manipulation × CS, $F<1$, NS; main effect of CS maintained, $F_{1,3}=31.69$, p=0.011) arousal. **B** Citalopram alone had no effect on cardiovascular (manipulation × CS, $F_{1,4}=1.171$, p=0.340; main effect of CS maintained, $F_{1,4}=19.39$, p=0.012) or behavioral (manipulation × CS, $F_{1,4}<1$; main effect of CS maintained, $F_{1,4}=30.29$, p=0.005) arousal.

Tables

Subject	Symbol	Cannulation target	Experimental history	NR. OF INFUSIONS (total)	
				sgACC/25	pgACC/32
Subject 1	□	sgACC/25, pgACC/32	-	16 (21)	5 (9)
Subject 2[i]	△	sgACC/25, pgACC/32	-	3 (3)	3 (3)
Subject 3	▽	sgACC/25	-	13 (16)	n/a
Subject 4	o	sgACC/25	-	10 (15)	n/a
Subject 5	◇	sgACC/25, pgACC/32	Aversive Pavlovian discrimination[ii]	17 (18)	4 (4)
Subject 6	⬡	sgACC/25, pgACC/32	Aversive Pavlovian discrimination[ii]	7 (11)	4 (6)
Subject 7[iii]	◑	sgACC/25, pgACC/32	-	3 (7)	0 (3)
Subject 8[iv]	☒	sgACC/25, pgACC/32	Resting-state, aversive Pavlovian discrimination[ii]	4 (9)	0 (6)
Subject 9[iv]	⊗	sgACC/25, pgACC/32	Resting-state, aversive Pavlovian discrimination[ii]	6 (11)	0 (4)[v]
Subject 10[vi]	▲	sgACC/25	-	2 (2)	n/a
Subject 11[vi]	▼	sgACC/25	-	2 (2)	n/a

Table S1. Experimental histories and number of infusions per site. Related to Table 1. The number of infusions (reported far right) includes the number of infusions contributing to the experiments reported here, together with the total number of infusions in brackets (including infusions for piloting DHK doses and infusions for experiments not reported here, carried out after the completion of experiments reported in this manuscript). [i]Due to a problem with implant cement, *Subject 2* lost her cannula implant during the early stages of the infusion protocol on the appetitive Pavlovian discrimination paradigm and was dropped from the study. [ii]*Subjects 5, 6, 8* and *9* had varying amounts of aversive Pavlovian discrimination training prior to the study. This involved 30-minute sessions five days a week, in which marmosets were presented with auditory cues which were paired either with a mildly aversive loud noise (0.3-0.7s, 115-118dB) or a neutral event (0.5s house-light off). *Subjects 5* and *6* failed to learn the discrimination and so testing was terminated and they were then transferred to the appetitive paradigm. *Subjects 8* and *9* additionally received infusions in an emotionally neutral resting-state condition prior to the study. [iii]*Subject 7* had telemetric probe failure early on during appetitive Pavlovian discrimination and so was moved onto PET imaging, for which cardiovascular measurements were not essential. [iv]*Subjects 8* and *9* had telemetry probe failures and so for these subjects, testing was conducted on appetitive paradigms which did not require cardiovascular measurements. [v]Unilateral cannula placement. [vi]*Subjects 10* and *11* took part in other studies following their human intruder test, the results of which are not reported here.

Infusion	Latency (seconds, mean ± SEM)	P value
sgACC/25 control	14.44 ± 2.35	
sgACC/25 DHK	23.36 ± 8.91	0.252
sgACC/25 CGP/LY	12.91 ± 2.95	0.485
sgACC/25 MB	20.13 ± 7.28	0.609
pgACC/32 control	15.05 ± 4.60	
pgACC/32 DHK	6.03 ± 1.49	0.244
pgACC/32 CGP/LY	13.75 ± 5.50	0.8
pgACC/32 MB	20.13 ± 7.28	0.776

Table S2. Consummatory (US+) latencies to start eating food reward. Related to Figures 2, S3, S5 and S6. P values reported from two-tailed paired *t*-tests comparing control (saline) sessions *vs.* drug sessions.

Locomotion values (s)	Subject						Mean ± SEM
	○	□	◇	▽	⬡	△	
Baseline							
Control	0.00	0.00	0.00	0.97	0.27	0.00	0.21 ± 0.16
sgACC/25 DHK	0.56	0.80	0.90	0.00	0.96	-	0.64 ± 0.17
sgACC/25 CGP/LY	0.00	0.00	0.00	0.00	1.92	1.73	0.61 ± 0.39
CS							
Control	1.33	1.90	0.35	0.62	1.23	1.04	1.08 ± 0.22
sgACC/25 DHK	1.34	0.93	1.91	0.71	1.21	-	1.22 ± 0.20
sgACC/25 CGP/LY	0.90	1.59	0.81	0.46	1.04	0.00	0.80 ± 0.22

Table S3. Assessment of locomotor activity during sgACC/25 control and over-activation infusion sessions. Related to Figures 2 and S3. Over-activation using DHK did not change baseline or CS locomotion (manipulation × phase, $F<1$, NS; main effect of manipulation, $F<1$, NS; main effect of phase, $F_{1,4}=19.83$, p=0.011). Over-activation using CGP/LY release did not change baseline or CS locomotion (manipulation × phase, $F_{1,5}=1.47$, p=0.279; main effect of manipulation, $F<1$, NS; main effect of phase, $F_{1,5}=2.78$, p=0.157). We additionally correlated CS-directed changes in MAP and CS-directed changes in locomotion across control, DHK and CGP/LY infusion types and found no evidence for a correlation (control, DHK and CGP/LY, $R^2=0.052$; not shown) supporting the notion that CS-directed changes in MAP are unrelated to alterations in locomotion.

Source of variation	ANOVAs on absolute MAP values compared to control						ANOVAs on head-jerk values compared to control					
	sgACC/25 DHK	sgACC/25 CGP/LY	sgACC/25 MB	pgACC/32 DHK	pgACC/32 CGP/LY	pgACC/32 MB	sgACC/25 DHK	sgACC/25 CGP/LY	sgACC/25 MB	pgACC/32 DHK	pgACC/32 CGP/LY	pgACC/32 MB
Manipulation (control *vs.* drug)	0.225	0.011	0.840	0.957	0.127	0.152	0.013	0.003	0.044	0.744	0.444	0.604
Phase (baseline *vs.* CS+)	<0.001	<0.001	<0.001	0.020	0.032	0.020	0.002	0.002	0.002	0.004	0.004	0.015
Manipulation × Phase interaction	0.045	0.029	0.900	0.660	0.575	0.704	0.006	0.003	0.128	0.633	0.444	>0.999
Post-hoc (baseline *vs.* CS+): Control	0.003	0.003	N/A				<0.001	<0.001	N/A			
Post-hoc (baseline *vs.* CS+): Drug	0.353	0.014					0.073	0.002				

Table S4. Analysis of variance (ANOVA) conducted on absolute MAP values and head-jerk values during baseline and CS+ periods for all appetitive Pavlovian infusion results. Related to Figures 2, S3, S5 and S6. Highlighted in grey are significant results at a threshold of $\alpha=0.05$. **MAP:** All comparisons showed a main effect of phase (indicating MAP values were significantly different between baseline and CS+ periods). Only sgACC/25 DHK and sgACC/25 CGP/LY infusions showed a manipulation × phase interaction, evidencing a differential effect on the baseline *vs.* CS+ period compared to control infusions. Post-hoc comparisons using Sidak's multiple comparisons test revealed that CS+ period absolute MAP did not significantly differ from baseline period values in the case of sgACC/25 DHK, indicating an abolition of appetitive anticipatory arousal. Whilst CS+ period absolute MAP values were still significantly different from baseline absolute MAP values in the case of sgACC/25 CGP/LY infusions, the magnitude of the difference was less than control infusions (control: 5.56 ± 0.86, CGP/LY: 2.66 ± 0.60, mean ± SEM; see **Fig. S3A**) consistent with a reduction but not abolition of anticipatory cardiovascular arousal. **Head-jerks:** All comparisons showed a main effect of phase (indicating head-jerk values were significantly different between baseline and CS+ periods). Only sgACC/25 DHK and sgACC/25 CGP/LY infusions showed a manipulation × phase interaction, evidencing a differential effect on the baseline *vs.* CS+ period compared to control infusions. Post-hoc comparisons using Sidak's multiple comparisons test revealed that CS+ period head-jerks did not significantly differ from baseline period values in the case of sgACC/25 DHK, indicating an abolition of appetitive anticipatory behavioral arousal. Whilst CS+ period head-jerk values were still significantly different from baseline head-jerk values in the case of sgACC/25 CGP/LY infusions, the magnitude of the difference was less than control infusions (control: 7.39 ± 0.50, CGP/LY: 3.50 ± 0.50, mean ± SEM; see **Fig. S3B**) consistent with a reduction but not abolition of anticipatory behavioral arousal.

Extracted factors	Percentage of variance explained	Behavior	Loading onto factor 1, representing anxiety
**1**	39.7317	Average height	0.816
2	14.3895	Bobs	0.769
3	10.2584	TSAB	0.688
4	9.0836	Tse egg calls	0.417
5	8.2543	Egg calls	0.332
6	6.9894	Tsik egg calls	0.323
7	4.0461	Tsik calls	-0.091
8	4.0321	Locomotion	-0.568
9	3.2148	TSAF	-0.790

Table S5. Exploratory factor analysis (EFA) and factor loadings of individual behaviors. Related to Figure 4. TSAB, time spent at back. TSAF, time spent at front. An EFA was carried out on the behavioral data of 171 marmosets undergoing the human intruder test as part of a screening protocol. Based on the point of inflection of a scree plot, a single factor (1) was identified accounting for 39.7% of variance. The pattern in which the individual behaviors load onto this factor suggest that it represents the marmosets' anxiety towards the intruder: a high factor score represents an animal high up and at the back of cage, far away from the intruder, remaining relatively still and performing a lot of head bobs, indicative of high anxiety.

Phase	Subject	Manipulation	TSAF, %	TSAB, %	Height, cm	Locm., %	Bobs	Tsik	Tsik-egg	Tse	Tse-egg	Egg
Baseline	◮	Control	23.48	0.00	24.13	4.75						
		Over-activation	14.70	0.00	25.67	7.09						
	⧩	Control	52.99	0.00	57.35	4.98						
		Over-activation	84.82	0.00	49.14	3.77						
	⊗	Control	91.95	2.23	29.77	8.34						
		Over-activation	72.96	12.81	37.71	7.48						
	Summary	Control mean	56.14	0.74	37.08	6.02						
		Control SEM	19.83	0.74	10.26	1.16						
		Over-activation mean	57.49	4.27	37.51	6.11						
		Over-activation SEM	21.67	4.27	6.78	1.18						
Intruder	◮	Control	61.94	9.82	45.35	8.6	4	0	0	0	0	0
		Over-activation	4.00	41.38	50.17	8.23	30	1	10	3	13	0
	⧩	Control	42.49	0.00	27.52	1.76	1	0	0	0	0	0
		Over-activation	8.02	31.02	68.73	4.51	1	4	3	0	0	0
	⊗	Control	38.29	0.86	43.64	9.03	10	0	1	0	4	2
		Over-activation	22.27	24.98	51.08	7.29	21	7	16	5	1	4
	Summary	Control mean	47.57	3.56	38.84	6.46	5	0	0	0	1	1
		Control SEM	7.28	3.14	5.68	2.35	3	0	0	0	1	1
		Over-activation mean	11.43	32.46	56.66	6.68	17	4	10	3	5	1
		Over-activation SEM	5.54	4.79	6.04	1.12	9	2	4	1	4	1

Table S6. Individual behavioral measures during the two-minutes prior to intruder exposure (baseline) and two-minute intruder period (intruder) across control and over-activation conditions. Related to Figure 4. TSAF, time spent at front. TSAB, time spent at back. Locm., locomotion. Marmosets do not exhibit any bobbing or tsik, tsik-egg, tse, tse-egg or egg vocalization behaviors during the baseline period, so these were not scored.

Manipulation	Hemisphere	sgACC/25 SUVR values by subject				Mean ± SEM
		□	▽	◇	◑	
Control	Left	0.856	1.028	0.843	0.822	0.887 ± 0.047
	Right	0.785	1.002	0.842	0.833	0.866 ± 0.047
Over-activation	Left	1.069	1.157	0.921	1.019	1.041 ± 0.049
	Right	1.023	1.040	0.933	0.992	0.997 ± 0.023
Over-activation + Ketamine	Left	1.017	0.974	0.882	0.807	0.920 ± 0.047
	Right	0.967	0.905	0.880	0.807	0.890 ± 0.033

Table S7. Measurements of SUVR changes across control, over-activation and [over-activation + ketamine] in an atlas-defined sgACC/25 ROI. Related to Figure 7. Within this ROI, there was a significant effect of manipulation on SUVR values (manipulation × hemisphere, $F_{2,6}<1$, NS; effect of manipulation: $F_{2,6}=6.22$, p=0.034). Planned comparisons using Fisher's LSD test revealed a significant difference between control *vs.* over-activation (p=0.016) and over-activation *vs.* [over-activation + ketamine] (p=0.037) conditions, but not for control *vs.* [over-activation + ketamine] (p=0.530) conditions.

Drug or drug cocktail	Mechanism	Route	Dose	Rate	Pre-treatment
Dihydrokainic acid (DHK)	EAAT2 antagonist	Central infusion	1.35µg/µL	0.5µL/min	10 minutes
CGP52432/LY341495 (CGP-LY)	$GABA_B$/$mGlu_{2/3}$ receptor antagonist	Central infusion	10ng/µL CGP52432 100pg/µL LY341495	0.5µL/min	15 minutes
Muscimol/Baclofen (MB)	$GABA_A$/$GABA_B$ receptor agonist	Central infusion	11.4ng/µL muscimol 0.214µg/µL baclofen	0.25µL/min	25 minutes
Ketamine	NMDA receptor antagonist	Intramuscular injection	0.5mg/kg	n/a	n/a
Citalopram	SSRI	Intramuscular injection	10mg/kg	n/a	30 minutes
Naloxone	Non-selective opioid receptor antagonist	Intramuscular injection	10mg/kg	n/a	10 minutes

Table S8. Mechanism, route of administration, dose and pre-treatment time for drugs used in the study. Related to Methods: *Drug treatments*. Pre-treatment refers to the time interval between completion of infusion and entry of the animal into the behavioral testing apparatus. All centrally administered drugs were infused over two minutes and injectors were left in place for one minute to facilitate adequate diffusion.
